# Genome-wide transcriptome profiling of transgenic hop (Humulus lupulus L.) constitutively overexpressing *Hl*WRKY1 and *Hl*WDR1 transcription factors

**DOI:** 10.1186/s12864-018-5125-8

**Published:** 2018-10-11

**Authors:** Ajay Kumar Mishra, Ganesh Selvaraj Duraisamy, Mudra Khare, Tomáš Kocábek, Jernej Jakse, Jindřich Bříza, Josef Patzak, Teruo Sano, Jaroslav Matoušek

**Affiliations:** 1 Biology Centre of the Czech Academy of Sciences, Institute of Plant Molecular Biology, Department of Molecular Genetics, Branišovská 31, 37005 České Budějovice, Czech Republic; 2 Department of Agronomy, Biotechnical Faculty, University of Ljubljana, Jamnikarjeva 101, SI-1000 Ljubljana, Slovenia; 3 Hop Research Institute, Co. Ltd., Kadaňská 2525, 43846 Žatec, Czech Republic; 4 Faculty of Agriculture and Life Science, Department of Applied Biosciences, Hirosaki University, Hirosaki, Aomori, 036-8561 Japan

**Keywords:** Bitter acids, Flavonoids, Genetic transformation, *Humulus lupulus*, Secondary metabolite, Transcription factors, Transcriptome analysis

## Abstract

**Background:**

The hop plant (*Humulus lupulus* L.) is a valuable source of several secondary metabolites, such as flavonoids, bitter acids, and essential oils. These compounds are widely implicated in the beer brewing industry and are having potential biomedical applications. Several independent breeding programs around the world have been initiated to develop new cultivars with enriched lupulin and secondary metabolite contents but met with limited success due to several constraints. In the present work, a pioneering attempt has been made to overexpress master regulator binary transcription factor complex formed by *Hl*WRKY1 and *Hl*WDR1 using a plant expression vector to enhance the level of prenylflavonoid and bitter acid content in the hop. Subsequently, we performed transcriptional profiling using high-throughput RNA-Seq technology in leaves of resultant transformants and wild-type hop to gain in-depth information about the genome-wide functional changes induced by *Hl*WRKY1 and *Hl*WDR1 overexpression.

**Results:**

The transgenic WW-lines exhibited an elevated expression of structural and regulatory genes involved in prenylflavonoid and bitter acid biosynthesis pathways. In addition, the comparative transcriptome analysis revealed a total of 522 transcripts involved in 30 pathways, including lipids and amino acids biosynthesis, primary carbon metabolism, phytohormone signaling and stress responses were differentially expressed in WW-transformants. It was apparent from the whole transcriptome sequencing that modulation of primary carbon metabolism and other pathways by *Hl*WRKY1 and *Hl*WDR1 overexpression resulted in enhanced substrate flux towards secondary metabolites pathway. The detailed analyses suggested that none of the pathways or genes, which have a detrimental effect on physiology, growth and development processes, were induced on a genome-wide scale in WW-transgenic lines.

**Conclusions:**

Taken together, our results suggest that *Hl*WRKY1 and *Hl*WDR1 simultaneous overexpression positively regulates the prenylflavonoid and bitter acid biosynthesis pathways in the hop and thus these transgenes are presented as prospective candidates for achieving enhanced secondary metabolite content in the hop.

**Electronic supplementary material:**

The online version of this article (10.1186/s12864-018-5125-8) contains supplementary material, which is available to authorized users.

## Background

Hop (*Humulus lupulus* L.) is a herbaceous, perennial climbing vine, a dioecious plant belonging to the *Cannabaceae* family, widely cultivated throughout the temperate regions of the world for the brewing industry as a source of flavour-active secondary metabolites, bitter acids, and shelf-life stabilizer. In addition, hop extracts and/or metabolome has received considerable attention in pharmaceutical applications due to their diverse biological properties, such as anti-carcinogenic [1], anti-inflammatory [2], estrogenic [3], sedative [4], antimicrobial [5] and antioxidation [6] activities. The female plants of hop produce cone-like inflorescences, commonly referred to as “hop cones” or “hops” contain a large number of highly metabolically active glandular trichomes (lupulin glands) on the inner side of bracts and bracteoles, which synthesize and/or secret specific secondary metabolites such as essential oils, bitter acids (humulone or α-acid and lupulone or β-acid) and prenylated flavonoids (xanthohumol and desmethylxanthohumol) during its phased maturation [7, 8]. The bracts represent the aggregation of modified leaves, which makes up the outer structure of female cones. In addition to cones, lupulin glands are also sparsely distributed on the undersides of leaves (Additional file 1: Figure S1), contain detectable levels of hop acids [9], terpenes [10], xanthohumol [11] and flavonols [12] and thus serve as the primary site of secondary metabolite accumulation [11]. Hop plants undergo different phenological growth stages and reaches peak maturity at around its third year of growth. Generally, after a three-year of normal growth, the hop cones completely develop and ripe with highest metabolome content [13].

**Fig. 1:**
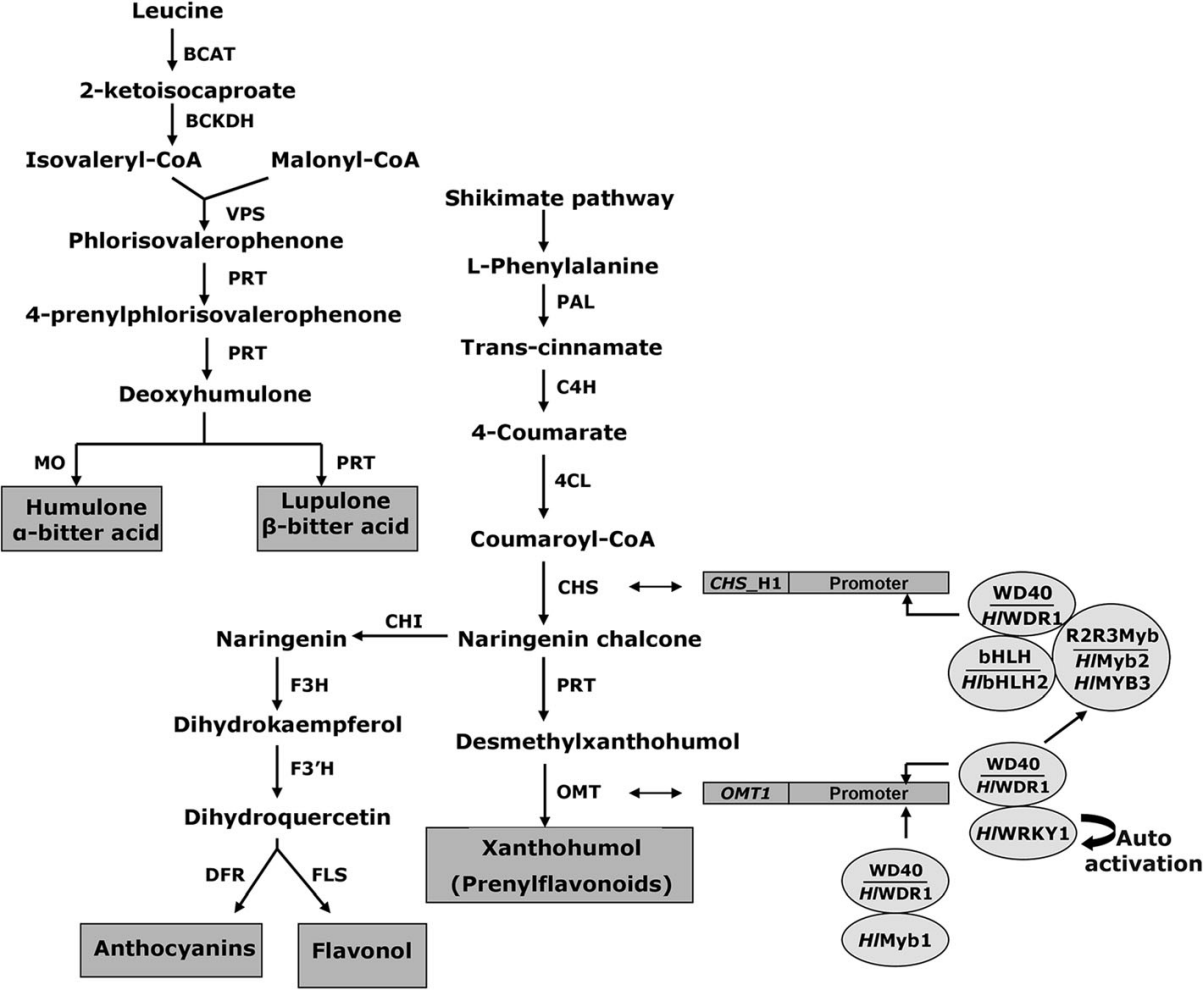
An overview of the bitter acid, phenylpropanoids and flavonoid biosynthesis pathways in the hop. The main intermediate compounds are shown with the abbreviation of respective enzymatic steps. Enzyme abbreviations are PAL: phenylalanine ammonia lyase; C4H: cinnamate 4-hydroxylase, 4CL: coumarate coenzyme A ligase, CHS: chalcone synthase, PRT: prenyltransferase; OMT: O*-*methyltransferases; VPS: valerophenone synthase, BCAT: branched chain aminotransferase; BCKDH: branched*-*chain alpha*-*ketoacid dehydrogenase; MO: monooxygenases; CHI: chalcone flavanone isomerase; F3H: flavanone 3-hydroxylase; F3′H, flavonoid 3′-hydroxylase; DFR, dihydroflavonol 4-reductase; FLS: flavonol synthase. *OMTI* and *CHS*_H1 represent gene isoforms of O*-*methyltransferases and chalcone synthase genes, respectively in the hop

The shikimate pathway serves as the primary source of biosynthesis of flavonoid and other phenylpropanoid precursors in plants. At the link between primary and secondary metabolism, phenylalanine ammonia-lyase (PAL) or tyrosine ammonia lyase (TAL) catalyze the non-oxidative deamination of phenylalanine to *trans*-cinnamate and direct the carbon flow from the shikimate pathway to the various branches of the general phenylpropanoids and flavonoids biosynthesis [14]. The other two enzymes cinnamate 4-hydroxylase (C4H), and 4-coumaroyl CoA-Ligase (4CL) catalyze other two committed successive steps leading to 4-coumaroyl-CoA substrate, which represents the prime branch point for all subsequent phenylpropanoid branches and flavonoid biosynthesis in plants [14]. The terminal step of prenylated flavonoids in hop cones is mediated by chalcone synthase CHS_H1 [15], prenyltransferase (PRT) [16] and O-methyltransferase 1 (OMT1) [11] enzymes with the involvement of several types of transcription factors, belonging to MYB, bHLH, WDR and WRKY families in either independent or combinatorial manner (Fig. 1) [17–20]. The parallel activation of *CHS*_H1 promoter has been shown to be driven by either highly organized ternary MBW complexes (*Hls*-Myb3/*Hl*bHLH2/*Hl*WDR1 or *Hl*MYB2/*Hl*bHLH2/*Hl*WDR1) or binary complexes (*Hl*bHLH2 /*Hl*WDR1) through protein-protein interactions (Fig. 1) [18, 19]. Recently we have cloned and characterized *Hl*WRKY1 (homolog of *At*WRKY75) transcription factor which forms a binary complex with WD40 repeat protein1 (*Hl*WDR1) and acts as a master transcription factor to activate structural genes of terminal steps and ternary MBW complex of the prenylflavonoid (PF) and bitter acids (BA) biosynthesis pathways [18]. The expression of *Hl*WRKY1 transcription factor can be activated by a protein kinase, modulated by autoactivation and dependent on RNA silencing machinery [19].

The *Hl*WRKY1 and *Hl*WDR1 transcription factors are preferentially expressed in lupulin glands, function as a master regulator to drive the PF and BA biosynthesis pathways [18, 19]. The homologs of these two transcription factors have multifunctional roles [21, 22]. In this context, it was imperative to understand whether constitutive expression of these two transcription factors could serve as an important strategy to enhance the PF and BA content by analyzing overall impact over the morphological, developmental attributes and other related pathways of hop. The several independent and cooperative hop breeding programmes around the world have been directed towards the development of new and improved cultivars with advantageous traits such as higher yield, enriched lupulin, high metabolome content to satisfy the demand of the brewing industry [13, 23]. However, conventional breeding methods have been largely constrained by the sexual incompatibility of developed lines, limited genetic resources, long cumbersome process and appearance of unintended characteristics [24]. Nevertheless, alternative direction involving the transformation of hop, utilizing either heterologous or homologous gene expression system may provide a promising approach for secondary metabolite engineering [25]. The genetic transformation technology, which is considered as an extension of conventional plant breeding technologies has been practiced in several plant species for the introduction of desirable agronomic traits from more than three decades and became an established technology to generate precise, rapid and stable modifications in utilized cultivars [26, 27]. Over the past years, genetic engineering technology has been used for manipulation of secondary metabolite biosynthesis in different plant species via constitutive overexpression of homologous and heterologous transcription factors [28–30].

The integration of transgene cassettes into the host genome could alter the expression of adjacent and downstream genes from the insertion site and owing to different feedback regulatory mechanism disruption of single gene activity alter the expression of hundreds or thousands of other genes [31]. Generally, genetically modified plants have been evaluated based on comparative metabolomics, proteomics and nutritional composition analyses [32]. Remarkably, a genomics and transcriptomics technique has been recommended by the European Food Safety Agency (EFSA) as an additional evaluation criterion to improve the breadth of comparative analysis [32].

In this regard, recent revolutionary advances in next-generation sequencing technologies, in conjunction with the refined computational tools provide rapid*,* cost*-*effective generation of transcriptomics resources and more accurate analysis of differentially expressed genes between transgenic plants and their wild-type equivalents [33]. Over the past decade, several studies were conducted based on high-throughput sequencing of plant transcriptome, which provided better and broader understanding of the molecular level information on various model and non model plant species such as olive [34], cucumber [35], chickpea [36], tomato [37], potato [38], sweet potato [39] and many more. These studies provided valuable molecular and genetic information, including the discovery of molecular marker and novel genes, insight into genetic network, transcriptional and posttranscriptional gene regulation and metabolic pathways to accelerate crop improvement program through breeding and genetic engineering techniques [40, 41]. To this end, the comparative transcriptome analysis serves as an integrated approach to provide insights into the molecular basis underlying specific biological events, profiling of changes in gene expression levels under specific experimental or environmental conditions and detect unique alternatively spliced isoforms of transcripts [42]. Moreover, the comparative transcriptome analyses have been widely used to examine the unintended pleiotropic effects in a number of transgenic plant systems by comparing them with their isogenic counterpart [43–46].

In this study, transgenic hop lines overexpressing binary transcription factors complex *Hl*WRKY1 and *Hl*WDR1 have been developed. The developed hop transgenic lines have been used further to understand the impact of transgenic events on transcriptome expression profiles in a leaf of red-bine Czech Osvald’72 cultivar of hop, which is well known for its unique metabolome content and composition. The comparative transcriptome analysis suggests that the overexpression of *Hl*WRKY1 and *Hl*WDR1 leads to upregulation of transcription factors and structural genes involved in PF and BA biosynthesis pathways, including differential regulations of genes involved in diverse biological processes in the hop. To our knowledge, our study represents one of the first pioneering efforts to enhance the expression level of genes of PF and BA biosynthetic pathway in the hop with significant evidence at the transcriptional level that genetically modified hop is not harmful concerning the biosafety of genetically modified organisms (GMOs).

## Methods

### Hop transformation and screening of transgenic lines

**Fig. 2:**
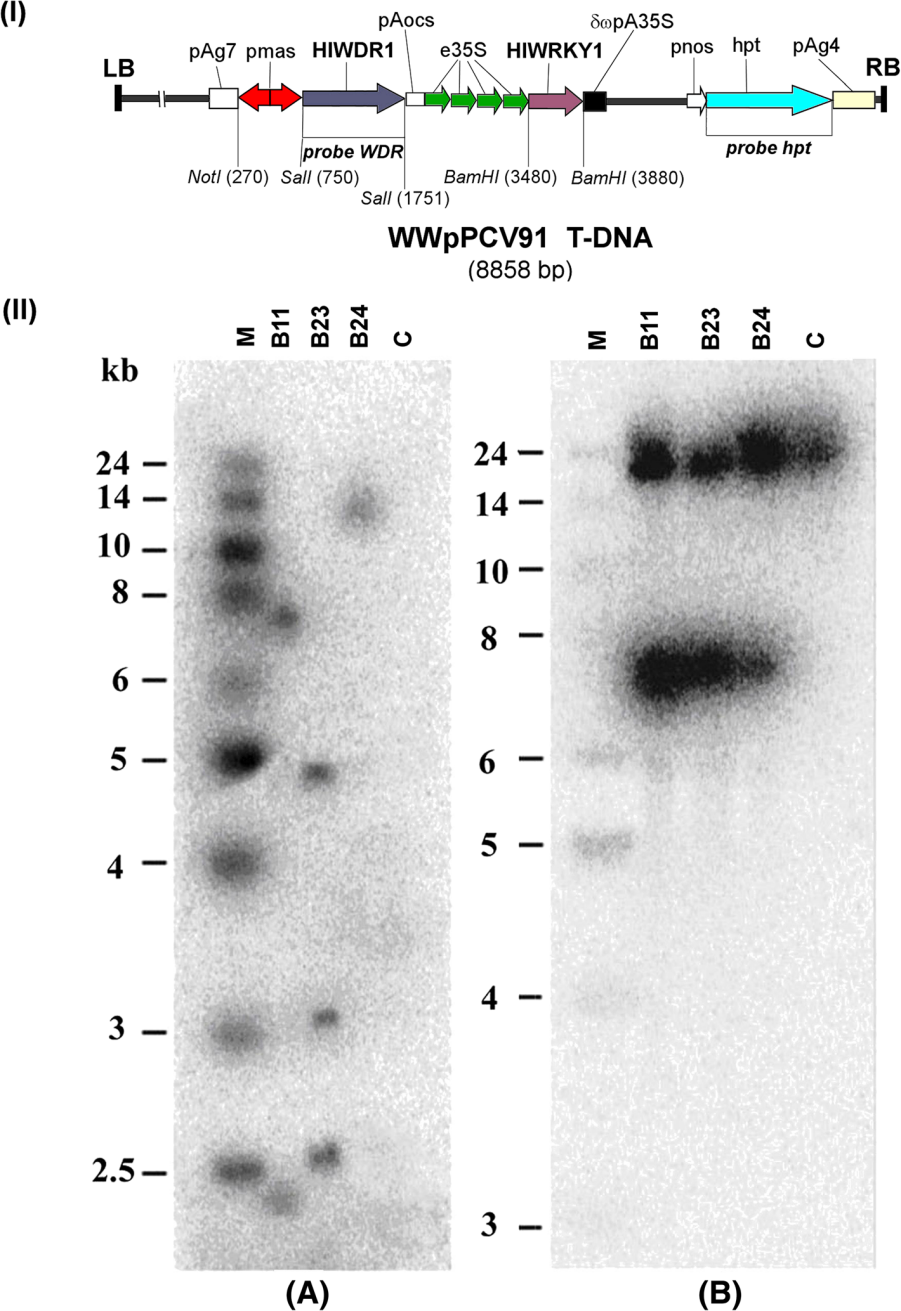
Molecular analysis of hop transgenic lines overexpressing *Hl*WRKY1 and *Hl*WRD1 transcription factors. (I) Schematic representation of the T-DNA region of the plant expression vector WWpPCV91, used for hop transformation.LB: left border, pmas: mannopine synthase bidirectional promoter, HPT: hygromycin phosphotransferase gene, e35S - enhancer of the 35S promoter from CaMV virus, pnos: nopaline synthase promoter, RB: right border. (II) Southern blot of hop genomic DNA isolated from WW-transgenic lines (B11, B23, B24) and control plants. The hybridization was performed with HPT (**a**) and WDR probe (**b**). The DNA markers (1 kb ladder, BRL) are positioned on the left sides

The full-length gene sequence of *Hl*WRKY1 (GenBank accession no: FR751557) and *Hl*WDR1 (GenBank accession no: FN689721) were amplified from our previously constructed lupulin-specific cDNA library using gene-specific primers (Additional file 2: Table S1) and cloned into dual expression cassette vector WWpPCV91 using adapter ligation-mediated PCR strategy as described previously [19]. The generated construct harboring *Hl*WRKY1 cDNA was fused to the tetramer of enhancer of *cauliflower mosaic virus* (CaMV) 35S promoter and *Hl*WDR1 cDNA was fused to mannopine synthase bidirectional promoter, respectively (Fig. 2) and was transferred into the *A. tumefaciens* strain GV3101 by electroporation [47]. The single colony of *A. tumefaciens* with the plasmid WWpPCV91 was inoculated in liquid Luria-Bertani medium (LB; 0.5% NaCl, 1% yeast extract and 1.6% tryptone) supplemented with 100 mg l^− 1^ carbenicillin and 50 mg l^− 1^ kanamycin. The bacterial cultures were grown until the optical density reached at 600 nm (OD_600_) of 0.6 at 28 °C with 200 rpm. The culture was diluted 1:50 into fresh LB medium with 200 μM acetosyringone (AS) and incubated at 28 °C until an OD_600_ of 0.6. The bacterial suspension was centrifuged at 6000 g for 10 min at 4 °C and bacterial pellet was suspended in MTA medium (10 mM MgSO_4_, 0.1% Tween 20, 200 μM AS) with an OD_600_ of 1.0 for transformation. The in vitro-derived internode stem segment explants (5–100 mm in length) of hop (cv. Osvald’s 72) were submerged into the bacterial suspension for 20 min. The infected explants were washed with sterile water and after wiping excessive water, the explants were cultured onto regeneration R medium (Murashige and Skoog basal medium containing 20 g l^− 1^ glucose, 1.0 mg l^− 1^ zeatin, 0.25 mg l^− 1^ IAA, 6 g l^− 1^ plant agar) supplemented with 200 μM AS for 3 days at 22–24 °C. Afterward, explants were transferred onto R medium containing 250 mg l^− 1^ Timentin and 1.5 mg l^− 1^ hygromycin B and were cultured under 16 h photoperiod at 22–24 °C for 4–6 weeks, until the ostentation of shoots. Regenerated shoots (Additional file 3: Figure S2A) were transferred to a rooting S medium (Murashige and Skoog basal medium supplemented with 20 g l^− 1^ glucose, 250 mg l^− 1^ Timentin, 1.5 mg l^− 1^ hygromycin B, 6 g l^− 1^ plant agar) impregnated with exogenous hormone supplements of IAA (1.0 mg l^− 1^) and IBA (1.0 mg l^− 1^) following previously described protocol [48]. To screen putative transgenic lines (WW) co-expressing *Hl*WRKY1 and *Hl*WDR1 transcription factors, Southern blot and PCR analyses were performed. Genomic DNA was extracted from leaves of WW-transformant and wild-type (WT) hop plant using a previously described protocol [49]. For the genomic DNA hybridization analysis, 15 μg of genomic DNA was digested overnight either with *Bgl*II or *Pac*I according to supplier’s instruction (New England Biolabs, MA, USA), separated on a 1% agarose gel at 25 V overnight with TBE buffer and transferred onto a positively charged nylon membrane (Qiabrane Nylon Plus) according to the manufacturer’s specifications (Qiagen, Hilden, Germany). The DNA probes (HPT and *Hl*WDR1) were radiolabelled with α-^32^P-labelled dCTP using Rediprime™ random prime labeling kit (Amersham Pharmacia Biotech, Freiburg, Germany) according to the manufacturer’s protocol. Pre-hybridization and hybridization reactions were carried out at 65 °C following a previously described protocol [50]. Membranes were analyzed by autoradiography (Typhoon 9200 PhosphoImager, USA). The PCR analysis was performed using WWpPCV91 vector specific forward and gene-specific reverse primer (Additional file 2: Table S1).

The independent WW-transformant and WT-lines were transferred from in vitro to in vivo growth chamber conditions at 22 °C with a 16 h photoperiod for gradual acclimatization and to ensure their survival (Additional file 3: Figure S2B). The successfully acclimatized three-month-old well rooted three WW-transformants (B11, B23, and B24) and wild-type (WT) plantlets were transferred to larger pots containing soil-vermiculite mixture (3:1) and grown under greenhouse conditions (Additional file 3: Figure S2C). The visual observations were taken regularly to evaluate growth and morphological characteristics (height, number of nodes, lupulin glands density, and leaf morphology). In addition, chlorophyll content was evaluated according to Lichtenthaler and Wellburn method [51]. The absorbance of the extract recorded at 646 and 663 nm wavelengths by using UV- spectrophotometry (Shimadzu, Japan).

### RNA extraction, high-throughput RNA sequencing and assembly generation

The leaves were harvested in the second year growing season of WW-transformants and WT-hop plants (28 months after the shifting to the greenhouse) for total RNA preparation. Total RNA was extracted from 100 mg of young leaves of control and three screened WW-transgenic lines (B11, B23, and B24) using Concert™ Plant RNA Purification Reagent (Invitrogen), followed by RNA purification and DNA contamination removal using DNA-free™ DNA Removal kit (Ambion, USA) according to the manufacturer’s instruction. The concentration of total RNA was measured by NanoDrop 2000 spectrophotometer (Thermo Scientific, USA), while quality assessment for the integrity of RNA samples was confirmed by Agilent Bioanalyzer 2100 RNA Nano chip (Agilent, USA) using RNA 6000 Nano assay kit (Agilent, USA). The isolation and enrichment of poly(A)-containing mRNA was performed from 5 μg total RNA of each samples using Dynabeads mRNA Purification Kit (Life Technologies, USA) and further used for cDNA synthesis using cDNA Synthesis System (Roche, Basel, Switzerland) as per the manufacturer’s protocol. The cDNA samples were sheared via nebulization into small fragments and were used for library construction using TruSeq RNA Library Preparation Kits. The resulting libraries were then paired-end sequenced (2x101bp) on an Illumina HiSeq2000 platform (Illumina) using myGenomics (Atlanta, USA) sequencing services. The raw sequencing data were subjected to removal of Illumina adapter sequences and quality filtering for empty reads and low-quality sequences (reads with unknown “N” sequences) using Trimmomatic v0*.*30 program [52]. The short reads below than 50 bases were dropped to exterminate the sequencing artifacts and the quality of reads was evaluated using FASTQC toolkit [53]. The high-quality reads were de novo assembled using CLC genomics workbench (v.10.0.1) with default parameters (mismatch cost *=* 2; insertion cost *=* 3; deletion cost *=* 3) into unique transcript sequences, termed as unigenes. All assembled unigene sequences were queried against the hop transcriptome database of HopBase genomic resources repository (http://hopbase.cgrb.oregonstate.edu/) using MEGABLAST at E-value <1e^− 3^, with a cutoff of percentage identity more than 95% and alignment length greater than 100 bp. The unigene sequences were also aligned to the hop draft genome assembly [54] using Spaln2 program [55]. The quantitative assessment and completeness of assembled unigenes was performed using BUSCO (Benchmarking Universal Single-Copy Orthologs; version 3.0) software [56].

### Functional annotation of the unigenes

The assembled sequences were aligned against the NCBI non-redundant (nr) protein database using BLASTX at E-value cut-off of 1.0 E − 3. Blast homology searches and homology-based functional annotations were performed using Blast2GO command line version 1.3.0 tool (https://www.blast2go.com/) [57]. Gene Ontology (GO; http://www.geneontology.org/) terms describing the biological process, molecular function, and cellular component were assigned to the unigenes through Blast2GO. The unigene sequences were also aligned to the Clusters of Orthologous Group (COG) database to predict and classify functions [58]. The single-directional best hit (SBH) method was used for KEGG (The Kyoto Encyclopedia of Genes and Genomes) pathway assignment of the assembled sequences using the online KEGG Automatic Annotation Server (KAAS; http://www.genome.jp/kegg/kaas/) to gain an overview of the gene pathway networks [59]. The PlantTFcat online tool was used for the identification of unigenes encoding transcription factor [60].

### Identification of differentially expressed genes and their network and pathway analysis

After assembly and annotation, the expression levels of each unigene between WT and WW-transformant lines were calculated by mapping clean read sets on the reference transcriptome as FPKM value (Fragment per kilobase of transcripts effective length per million fragments mapped to all transcripts) by expectation-maximization (RSEM) protocol using in-built scripts in the Trinity software package [61]. The normalization of the data among different libraries was performed using the Trimmed Mean of M-values normalization method in Trinity. The obtained count value was exported to Bioconductor software package DESeq2 [62] to identify the differentially expressed gene transcripts (DEG) using the Benjamini-Hochberg false discovery rate (FDR) < 0.05. The expression of a particular sequence was considered significantly different when the adjusted *P*-value obtained using this method was ≤0.05 and there was at least a two-fold change (≥2 or ≤ − 2) in the sequence count between WT and WW-libraries. The FPKM values for each transcript were log-transformed and normalized, which was subsequently used for calculation of matrix distance with Euclidean distance and complete-linkage methods. The R statistics package heatmap3 [63] was used for construction of heatmap. The DEGs were used for GO terms/KEGG pathway enrichment analyses using hypergeometric test equivalent to one-tailed Fisher’s exact test with a FDR value of 0.05 using the AgriGO toolkit [64], functional analysis and pathway visualization was performed using MapMan tool [65]. Protein families were assigned to DEGs by searching them against the Protein family (Pfam) database using HMM-based tool pfamscan [66].

### Validation using real-time quantitative RT-PCR

The differential expression of eight structural and six regulatory genes associated with phenylpropanoids and flavonoids biosynthesis pathway was subjected for a real-time quantitative reverse transcriptase PCR (qRT-PCR) validation of the transcriptome data. The first strand cDNA was synthesized from 5 μg of aliquots of the total RNA extracted for sequencing as described earlier using Superscript® III First-strand cDNA Synthesis system (Invitrogen, USA) according to the manufacturer’s instructions. Each qRT-PCR mixture consisted of 200 ng first strand cDNA, 10 μl SYBR Green Real-Time PCR Master Mix (Invitrogen, USA), 10 pmoL of forward and reverse gene-specific primers (Additional file 2: Table S1). Additionally, primers specific to hop *GAPDH* gene were used as an endogenous control to normalize the expression level of each gene [67]. PCR amplification was performed in an IQ5 Real-Time PCR Detection System (Bio-Rad, CA, USA) under the following conditions: 95 °C for 10 min, followed by 40 cycles at 95 °C for 15 s and at 60 °C for 1 min. The melting curve analysis was performed to assess the specificity of the PCR primer specific product by maintaining the reaction at 95 °C for 1 min, cooling the sample to 55 °C for 1 min and further heating to 95 °C at a rate of 0.5 °C per 6 s. The relative expression levels (fold-change) of the selected genes were calculated using the comparative Ct (2^−ΔΔCt^) method [68]. For each sample, the experiment was carried out in three independent technical replicates and based on that error bars were calculated.

### Analysis of polyphenols and flavonoids content in WW-transgenic line

The extract preparation (methanol: H_2_O) from dried leaf samples of WT and WW-transgenic plants, HPLC analysis of total phenolics and flavonoids content were performed as described previously [17, 69].

## Results

### Molecular, biochemical and morphological analysis of WW-transgenic lines

For this study, we have selected three independent hygromycin resistant WW-transgenic lines (B11, B23, and B24) generated using *Agrobacterium-*mediated transformation methods. The WWpPCV91 plasmid-based plant expression vector harboring dual expression cassettes (Fig. 2a) was used to create these transgenic lines. Southern blot analysis using *HlWDR1* and hygromycin phosphotransferase (*HPT*) gene confirmed that T-DNA was stably inserted into chromosomal DNA (Fig. 2b). The qRT-PCR of the regenerated plantlets (3-month-old) confirmed the presence and overexpression of transgenes in WW-lines (Additional file 4: Figure S3). The plants were transferred to the greenhouse and monitored throughout their vegetative development phase (year: 2016–2017) with special emphasis on plant morphology and growth. The growth performance of WW-transgenic lines was observed to be superior to WT-plants with much larger immature leaves of comparable position on plants of the same age (Additional file 5: Table S2; Additional file 3: Figure S2D). The chlorophyll content in WW-transgenic plants was higher than WT, suggesting the better photosynthetic performance of WW-lines (Additional file 5: Table S2). The lupulin gland distribution in transgenic lines was almost similar to that WT with respect to the differences of the epidermal cell size.

### Sequencing and de novo transcriptome assembly analyses

**Fig. 3:**
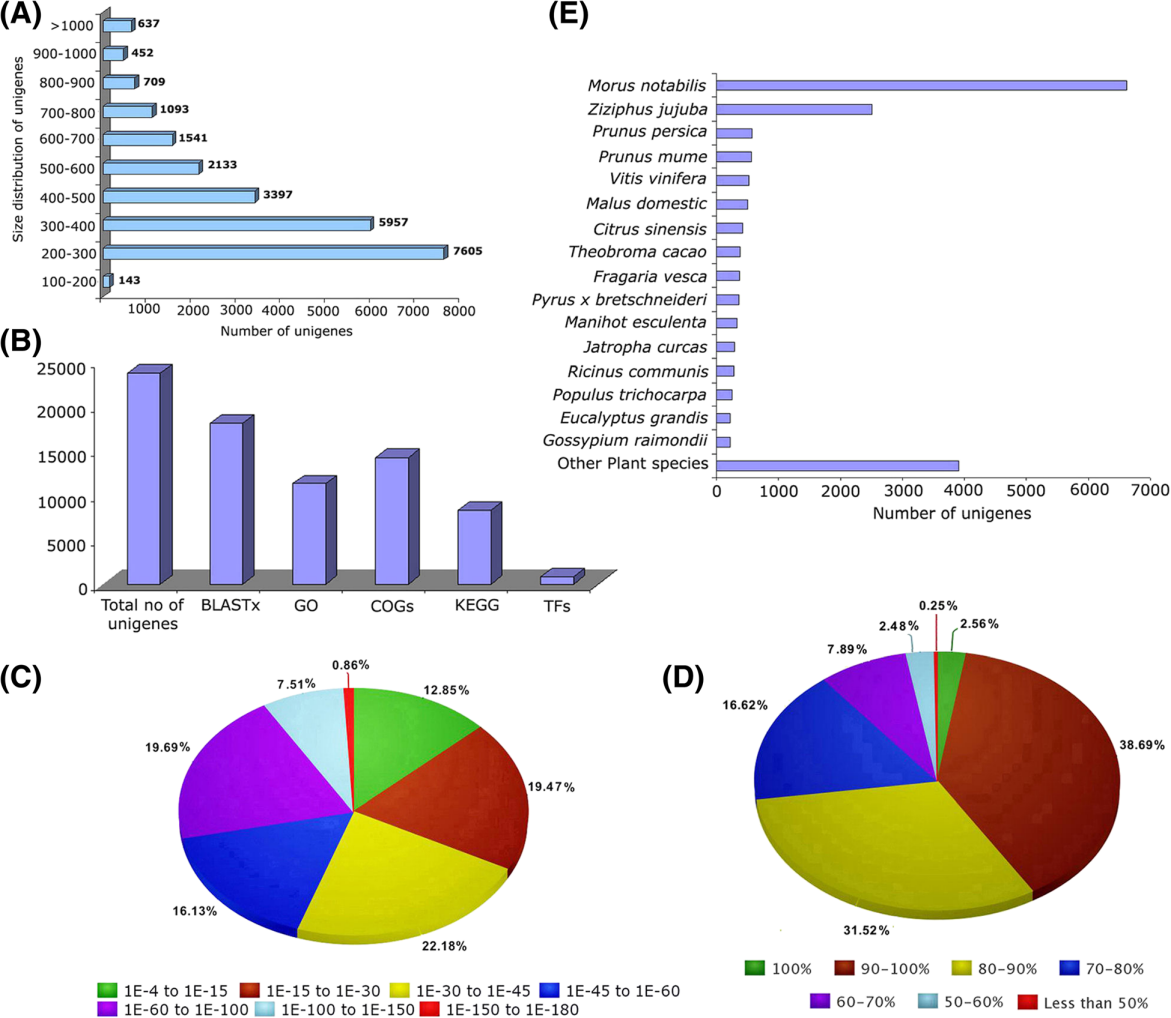
Characteristics of assembled unigenes. **a** Length distribution of assembled unigenes; **b** Annotation statistics of assembled unigenes; **c** E-value distribution of the BLASTX hits against the nr protein database for each unigene with a cutoff E-value of 1.0 E^− 3^; **d** Similarity distribution of the top BLASTx hits for each unigene; **e** BLASTx top-hit species distribution of unigenes

To obtain a comprehensive overview of the gene expression pattern in WW-transgenic lines, RNAseq libraries were constructed from the leaf tissue from individual two independent transgenic (B11 and B23) and WT (Osvald 72 cv.) lines with their technical replicates. High throughput sequencing run generated over 29 and 32 million raw reads in WT and WW samples, respectively (Table 1). After the removal of the adaptor and filtering out the low-quality reads at high stringency using Trimmomatic software, over 24 and 26 million high-quality reads were obtained for WT and WW groups, respectively. The transcriptome datasets (raw data) generated in this study have been deposited at the Sequence Read Achieve (SRA), National Centre for Biotechnology Information (NCBI) with the accession numbers SRR6308266, SRR6308267, SRR6308195 and SRR6308265 as biological replicates for WT and WW, respectively. All clean reads were subjected to the de novo assembly, which resulted in a total of 23,666 unigenes with size ranging from 157 bp to 2002 bp with an average unigene size of 436 bp (Table 1). The average unigene size was much longer (436 bp) than those identified in previous studies in *Withania somnifera* (200 bp) [70], *Ipomoea batatas* (202 bp) [71], *Eucalyptus grandis* (247 bp) [72] and *Physalis peruviana* (371 bp) [73]. The N50 of 452 bp value was obtained for the current transcriptome assembly and 9962 unigenes (42.10%) were longer than 400 bp (Fig. 3a). The average GC content of hop unigenes was 41.10%, which was comparable to the GC levels of unigenes of *Sophora flavescens* (39.9%) [74], chickpea (40.3%) [75], *Spinacia oleracea* (42.5%) [76] and *Glycine max* (43%) [77]. The comparative analysis of unigene sequences to hop transcriptome database available in HopBase showed that a total of 21,545 (91.03%) matched with greater than 95% sequence identity, indicated the broad representation of our unigenes. Approximately 18,565 (78.45%) unigenes were mapped to the draft hop genome assembly, which could be due to the incompleteness of the genome sequence, level of genetic variation between cultivars and the existence of large amounts of intergenic noncoding RNAs etc. [78], suggesting that our unigenes could serve as a valuable complementary resource for hop genomics.

**Table 1:** Statistics of RNA-seq analysis and assembly for hop

Item	Library	Number	Total Bases (GB)
Raw read	WT	29,177,758	2.20
	WW	32,818,870	2.50
Clean read	WT	24,399,800	1.79
	WW	26,889,520	1.96
Average Length (bp)	WT	422	
	WW	416	
Unigenes			
No. of Unigenes (n)		23,666	
Average Length (bp)		436	
Maximum Length (bp)		2200	
Minimum Length (bp)		106

### Functional annotation and classification of hop transcriptome sequences

Functional annotation of the assembled sequences against the NCBI non-redundant (nr) protein database showed that 18,048 (76.26%) matched to the nr protein database, whereas 5618 (23.74%) did not exhibit significant homology with sequences in the nr database (Additional file 6: Table S3, Fig. 3b). The E-value distribution of aligned unigenes against nr database showed that 60.90% of unigenes had an E-value of less than 1.0 E^− 50^ (Fig. 3c). The similarity distribution of the aligned unigenes compare to sequences in the nr database illustrated that 41.25% of unigenes had significant homology higher than 90%, followed by 58.51% of the sequences with homology between 50 and 90%; whereas only 0.25% of the sequences had homology lower than 50% (Fig. 3d). Furthermore, the species distribution analysis of unigenes based on their BLASTx alignment against the nr protein database showed that approximately 47.82% of total unigenes were matched with sequences from six dicotyledonous species, namely, *Morus notabilis* (27.96%), *Ziziphus jujuba* (10.61%), *Prunus persica* (2.46%), *P. mume* (2.41%), *Vitis vinifera* (2.21%) and *Malus domestica* (2.15%) (Fig. 3e). The annotation information for the majority of unigenes based on their homologous matches was obtained, which demonstrates the high accuracy of assembled transcript sequences. BUSCO analysis against a core set of 1440 single*-*copy orthologous genes of plants indicated the presence of 62% as complete, 26% fragmented, and 12% missing orthologs in our de novo assembly. The functional classification of unigenes based on BLAST search against the gene products in the GO database classified 14,184 matched unigenes into the three main GO categories, including 40 functional groups (Fig. 3b). A total of 72,581 GO functional terms was obtained, among them the biological process comprised the major category (35,254, 48.57%) followed by cellular component (22,022, 30.34%) and molecular functions (15,305, 21.09%) (Additional file 7: Figure S4A). Consistent with our observations, the prominence of the biological process over the molecular function and cellular components under the GO categories have been reported in other plant species such as cucumber [35], sesame [79], wheat [80], spinach [76] and many more.

It was found that 16,386 of the total unigenes have significant homology in the COG database (Fig. 3b) with multiple functions resulting in 74,723 functional annotations and these unigenes were classified into 25 categories (Additional file 8: Figure S5A). Of these categories, “general functional prediction only” (12,123, 16.22%) represented the largest group, followed by “inorganic ion transport and secretion” (10,128, 13.55%), “amino acid metabolism and transport” (7136, 9.55%), “post-translational modification, protein turnover, chaperone function” (6367, 8.52%), whereas the smallest groups were assigned to “nuclear structure” (53, 0.07%), followed by “cell motility” (112, 0.15%) and “extracellular structures” (134, 0.18%) (Additional file 8: Figure S5A).

To further investigate the biological functions of putative proteins and their biochemical pathways, the 8271 KEGG annotated unigenes (Fig. 3b) were grouped into five different functional groups (Table 2). KEGG pathway analysis annotated largest number of unigenes against “metabolism” with most of them represented “carbohydrate metabolism” (5.78%), “amino acid metabolism” (4.33%), “lipid metabolism” (3.64%), “energy metabolism” (3.54%), “biosynthesis of secondary metabolites” (1.73%) and other sub-categories. Strikingly, classified unigenes into the category “biosynthesis of secondary metabolites” were found to be linked with various secondary metabolites associated pathways, such as sesquiterpenoid and triterpenoid biosynthesis, flavonoid biosynthesis, and prenylflavonoids biosynthesis and many more. This observation was consistent with the previous notion that most of the secondary metabolite biosynthesis related genes are expressed in hop leaf tissues at detectable levels [11]. A total of 4781 unigenes were annotated into pathways related to “genetic information processing” included genes involved in transcription, translation, replication and repair, and protein folding, processing, and degradation. In addition, unigenes were also classified into pathways related to “cellular processes” and “environmental information processing” which accounted for 1676 and 1565 unigenes respectively of the KEGG annotated sequences (Table 2).

**Table 2:** Classification statistics for unigenes (UG) and differentially expressed genes [up-regulated (UR) and down-regulated genes (DR)] in WW-transformant lines according to KEGG pathway analysis

KEGG categories	Number of			KEGG categories	Number of		
	UG	UR	DR		UG	UR	DR
Metabolism							
Carbohydrate Metabolism	772	20	3	Cellular Process			
Energy metabolism	473	6	6	Transport and catabolism	1011	11	8
Lipid metabolism	487	24	2	Cell growth and death	331	7	1
Nucleotide metabolism	210	1	3	Cellular community - eukaryotes	99	0	0
Amino acid metabolism	578	4	2	Cellular community - prokaryotes	83	4	0
Metabolism of other amino acids	221	9	4	Cell motility	152	2	0
Glycan biosynthesis and metabolism	349	5	1	Environmental information processing			
Metabolism of cofactors and vitamins	325	5	6	Membrane transport	599	19	4
Metabolism of terpenoids and polyketides	181	5	1	Signal transduction	779	18	4
Biosynthesis of other secondary metabolites	231	10	4	Signaling molecules and interaction	187	6	0
Xenobiotics biodegradation and metabolism	127	3	2	Unclassified			
Enzyme families	921	9	7	Metabolism	229	12	3
Genetic information processing				Genetic information processing	68	0	0
Transcription	744	5	3	Cellular processes and signaling	104	2	0
Translation	1574	10	12	Viral protein family	0	0	0
Folding, sorting and degradation	1703	9	10	Poorly characterized	69	0	1
Replication and repair	760	7	4				
RNA family	0	0	0				
				Total	13,367	213	91

A total of 840 unigenes, accounted for 3.55% of the transcriptome, were classified into 72 putative transcription factors families (Additional file 9: Table S4). Among the 72 transcription factors families, C2H2, WD40-like, Hap3/NF-YB, CCHC(Zn), GRAS, MYB-HB-like, WRKY, bHLH, AP2-EREBP, SET, bZIP, PHD families were the top 12 classes (Additional file 10: Figure S6).

### Identification of differentially expressed genes and functional analysis

To compare the gene expression levels in the WT and WW-libraries, FPKM values of assembled unigenes were calculated. The mapping of all the reads onto the non-redundant set of hop transcripts revealed that the number of reads corresponding to each transcript ranged from 0.04 to 1972.80 for WT (FPKM) and from 0.09 to 4896.07 (FPKM) for WW library, respectively, indicating a very wide range of expression levels of hop transcripts (Additional file 11: Table S5). Transcriptome comparison resulted in the identification of 522 differentially expressed genes (DEGs, *p* ≤ 0.05, logFC ≥2 or ≤ − 2), among them 385 were found to be significantly up-regulated whereas, 137 were significantly down-regulated in WW-transgenic line compared to WT hop plant. Approximately 89% of DEGs (349 up-regulated genes and 115 down-regulated) were annotated against the nr protein database of NCBI (Additional file 11: Table S5).

**Fig. 4:**
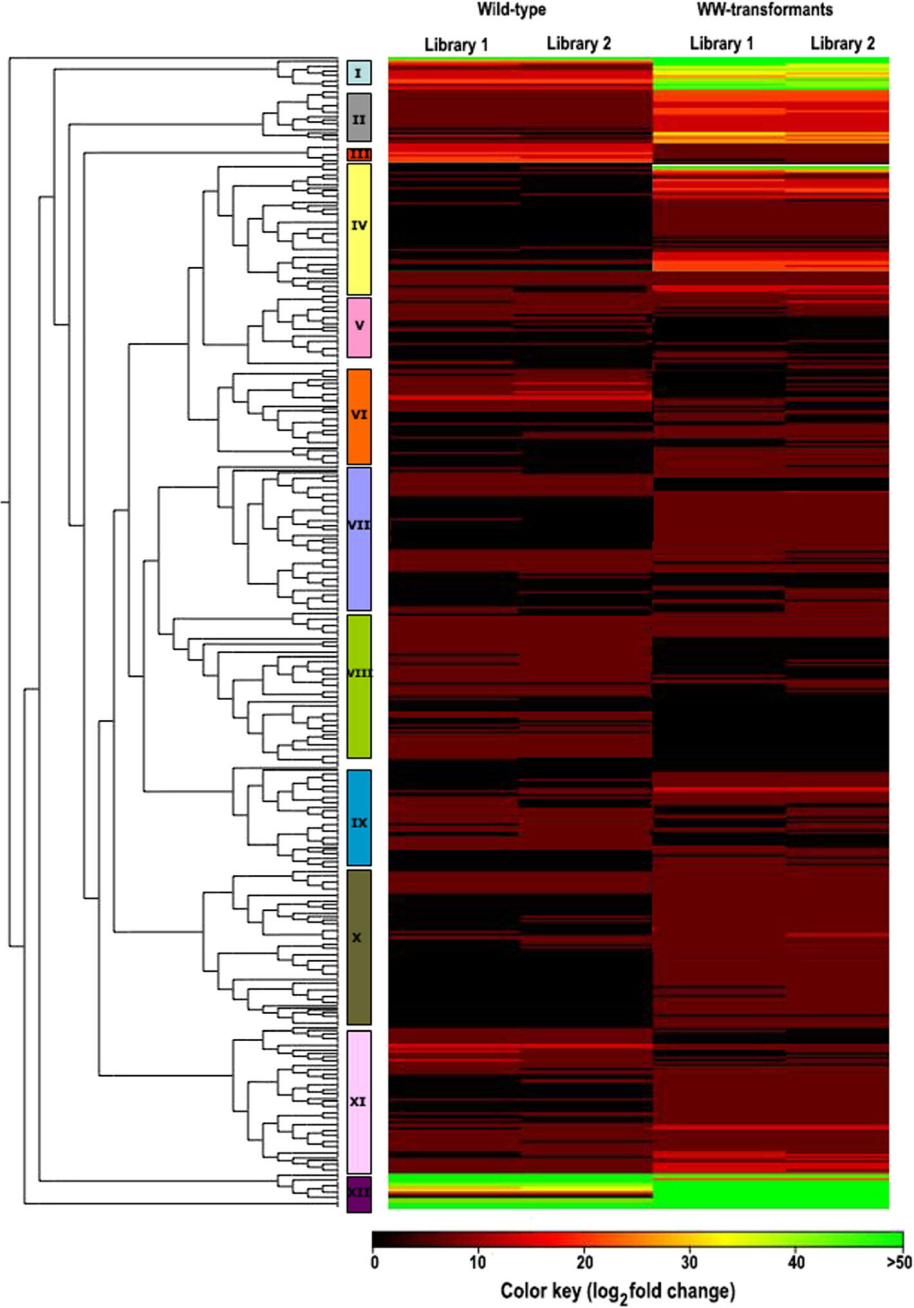
Heat map and complete linkage hierarchical clustering of differentially expressed genes in leaves of WW transformants compared with wild-type hop. Colors on vertical represent the clustered genes based on gene expression, the horizontal line represents the single gene and color of the line indicates the average gene expression in WW transformants. The signal ratios were shown in a black-green color scale, where green indicated high expression level and black indicated low expression level

Hierarchical cluster analysis based on FPKM values arranged 522 DEGs into twelve major clusters and were consistent within WT and WW-lines of hop (Fig. 4). Cluster I and II were enriched with unigenes encoded enzymes and transcription factors involved in the secondary metabolite synthesis, namely *PAL*, *C4H*, *4CL*, *CHS*_H1, *PRT1*, *OMT1 VPS*, flavanone 3-dioxygenase (*F3D*), Isoflavone 2′-hydroxylase (*I2H*), *Hl*WRKY1, *Hl*WDR1, *Hl*Myb2, *Hl*Myb3, *Hl*bHLH2. The genes grouped in cluster VIII were found to be down-regulated in WW transformants. Taken together, heat map results again reinforce the elevated level of expression of genes associated PF and BA biosynthesis pathway in WW transformants.

Functional categorization based on GO enrichment analysis using the Fisher’s exact test at the false discovery rate of 0.05 provided the statistically significant GO terms for DEGs. The GO annotation of DEGs categorized 424 unigenes into 30 functional groups, whereas, 98 unigenes were not classified (Additional file 7: Figure S4B). Among GO categories, “biological process” comprising the major domain of DEGs followed by “cellular component” and “molecular function”. Among the various biological process categories, functional enrichment analysis of DEGs revealed that genes involved in “cellular process” and “metabolic process” were significantly enriched (Table 3) and up-regulated (Additional file 7: Figure S4B). Strikingly, unigenes involved in growth, developmental process, and anatomical structure morphogenesis were found to be enriched suggested that some aspects related to these processes might have been activated in WW-transformant lines. The improved growth performance and larger leaf size of WW-transformant lines in optimal growth condition (Additional file 3: Figure S2D) could be correlated with these observations. In addition, GO terms related to “binding”, “catalytic activity”, “hydrolase activity”, “transferase activity”, “transporter activity” and “transcription factor activity” were enriched among “molecular function”, whereas within “cellular components” category “cell”, “cytoplasm”, “membrane”, “plastids” domains were enriched (Table 3). This observation suggested that these functions were enhanced in WW-transformant lines. Analysis of the COG classification of DEGs indicated their grouping in all COG functional categories except N (cell motility) category (Additional file 8: Figure S5B). The identified DEGs were subjected to KEGG pathway enrichment analysis. A total of 162 (42.08%) up-regulated and 67 (48.91%) down-regulated unigenes were annotated and assigned to 7 main categories (Table 2).

**Table 3:** Gene Ontology (GO) Functional Enrichment Analysis of differentially expressed genes in hop

GO ID	Ontology	Category	Number of DEGs in subgroup	Number of unigenes in subgroup	*P*-value	FDR
GO:0008152	metabolic process	P	161	10,614	8.30E-21	6.80E-19
GO:0009058	biosynthetic process	P	62	5118	0.00039	0.008
GO:0009987	cellular process	P	32	11,684	9.50E-07	3.20E-05
GO:0050896	response to stimulus	P	32	4057	0.53	1
GO:0006950	response to stress	P	24	2320	0.11	0.82
GO:0032502	developmental process	P	22	2304	0.21	1
GO:0048856	anatomical structure development	P	10	1726	0.88	1
GO:0003824	catalytic activity	F	176	9638	4.60E-34	2.30E-32
GO:0005488	binding	F	112	11,258	0.0025	0.031
GO:0016787	hydrolase activity	F	74	3478	2.90E-15	7.20E-14
GO:0016740	transferase activity	F	55	3321	1.50E-07	2.50E-06
GO:0005215	transporter activity	F	17	1473	0.081	0.5
GO:0003700	transcription factor activity	F	8	2173	1	1
GO:0005623	cell	C	129	15,217	0.16	1
GO:0016020	membrane	C	71	4068	1.40E-10	6.40E-09
GO:0005737	cytoplasm	C	48	6822	0.83	1
GO:0009536	plastids	C	15	2965	0.98	1

*P* Biological process, *F* Molecular function, *C* Cellular component

**Fig. 5:**
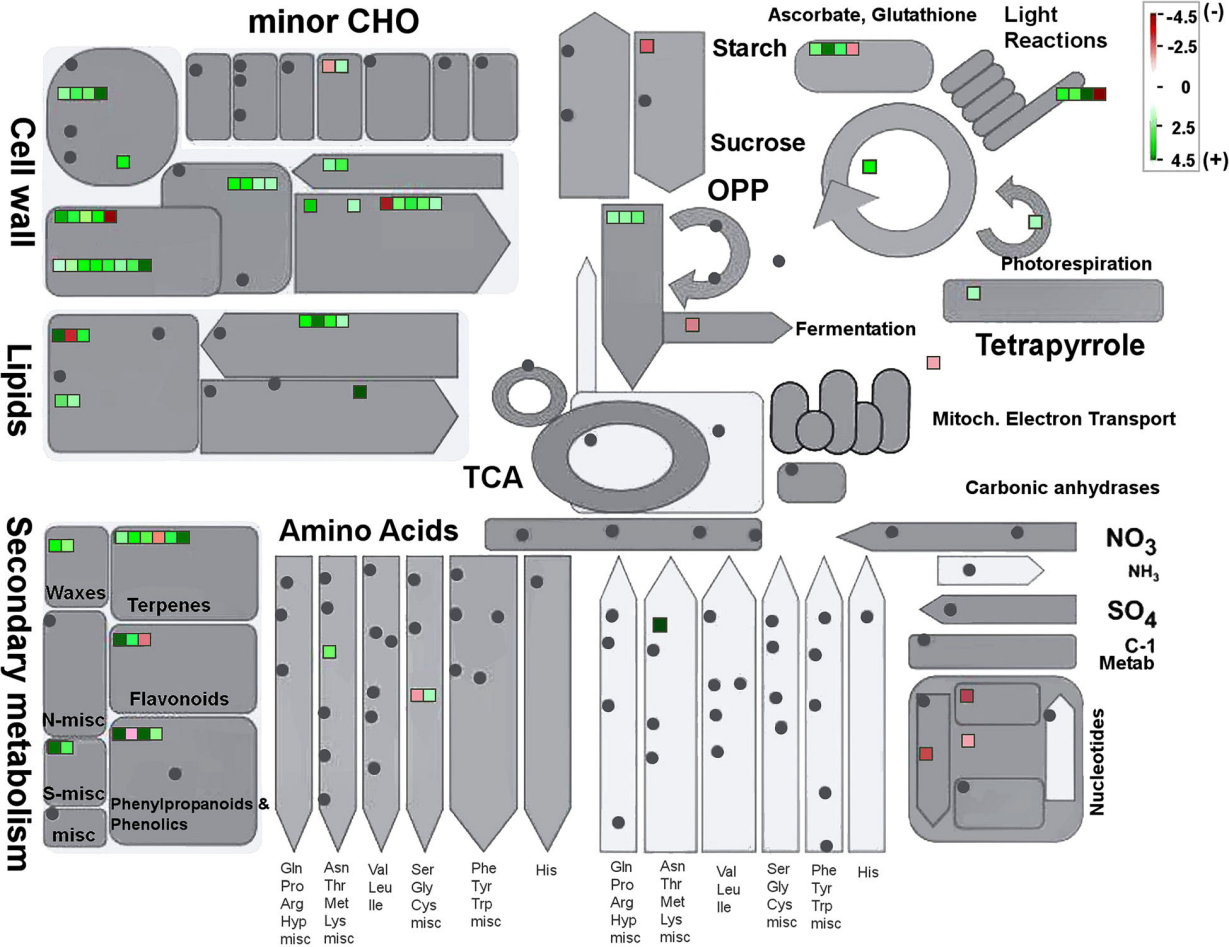
MapMan visualization of changes in transcript levels in WW-transformant compared with wild-type hop. The log_2_ fold changes of significantly differentially expressed genes associated with general metabolism were imported and visualized in MapMan. Red and green displayed signals represent a decrease and an increase in transcript abundance, respectively in WW-transformants (library 1: B11 and library 2: B23) relative to the wild-type of hop. The scale used for coloration of the signals (log_2_ ratios) is presented

Furthermore, DEGs were imported into MapMan for pathway-based analysis and visualization to gain an unbiased overview of important pathways or biological processes changed in WW-transformants. Consistent with GO analysis, DEGs associated with the secondary metabolite biosynthesis pathway showed upregulation (Fig. 5) in WW-transformants. Moreover, genes involved in cell wall metabolism, lipid metabolism, light reactions, and photorespiration were up-regulated, while those involved in starch degradation were down-regulated.

### Validation of secondary metabolite biosynthetic pathway associated DEGs by qRT-PCR

**Fig. 6:**
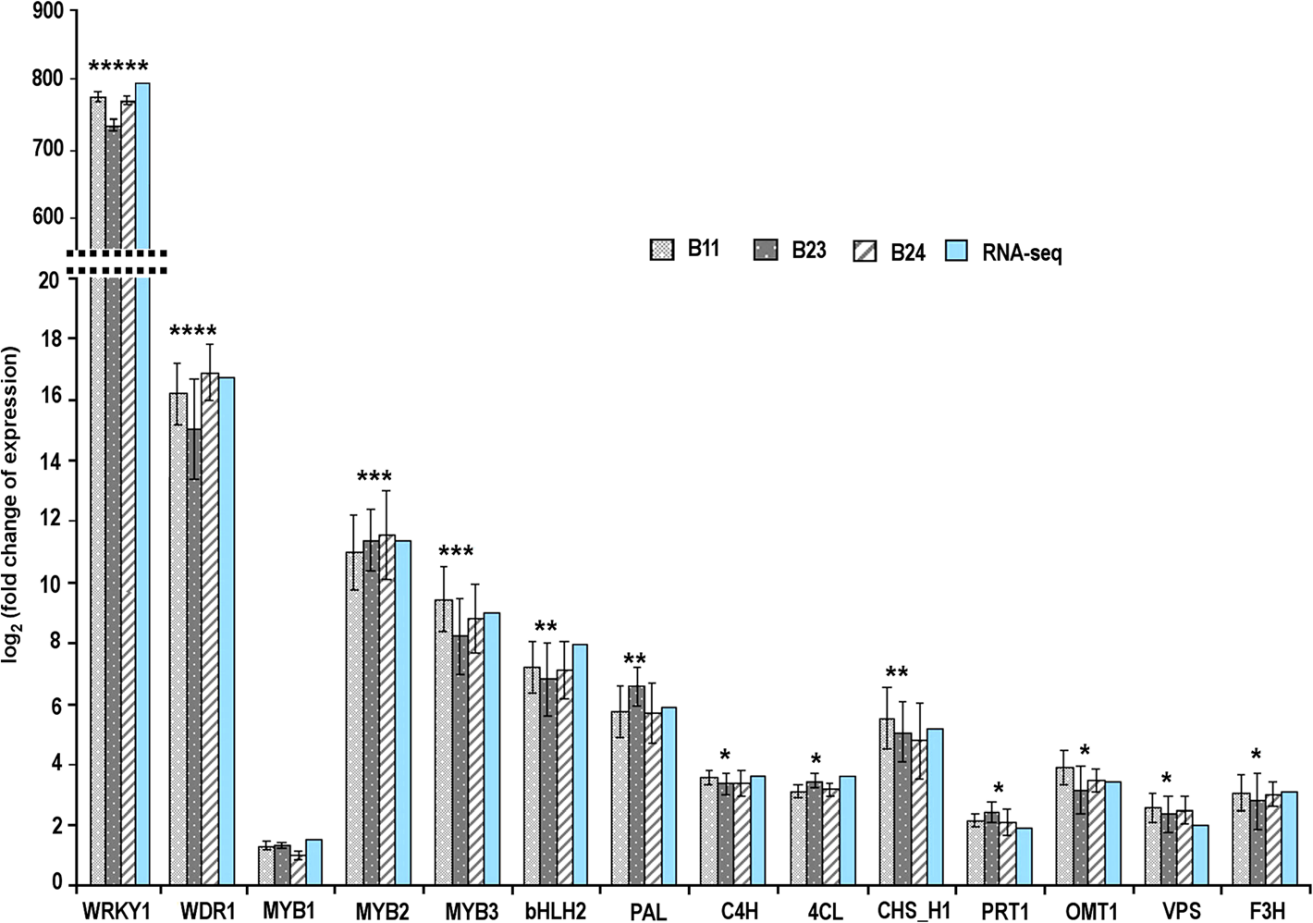
Validation of RNA sequencing by RT-qPCR. Graph showing fold change of the structural and regulatory genes in a leaf of hop among transgenic lines (B11, B22, and B23) overexpressing *Hl*WRKY1 and *Hl*WDR1 transcription factors. PAL: phenylalanine ammonia lyase; C4H: cinnamate 4-hydroxylase, 4CL: coumarate coenzyme A ligase, CHS_H1: chalcone synthase isoform 1, PRT1: prenyltransferase 1; OMT1: O*-*methyltransferases isoform 1; VPS: valerophenone synthase, F3H: flavanone 3-hydroxylase. qRT-PCR analyses were normalized using GAPDH as an internal control gene. The fold change of each gene was calculated by the 2^−ΔΔ*CT*^ method. *Statistically significant differences (*P* < 0.05); **significant at *p* < 0.01

Based on the result of Illumina sequencing data*,* the expression profiles of selective structural and regulatory genes involved in PF, BA and flavonol biosynthesis pathways were analyzed in leaves of WT and WW-transgenic hop lines using qRT-PCR. The real-time gene expression analysis suggested up-regulation of structural genes, namely *PAL*, *C4H*, *4CL*, *CHS*_H1, *PRT1*, *OMT1*, *VPS* and genes encoding regulatory proteins, namely *Hl*WRKY1, *Hl*WDR1, *Hl*Myb2, *Hl*Myb3, *Hl*bHLH2 (Fig. 6). The expression level of *Hl*WRKY1 and *Hl*WDR1 was comparable in all WW-transgenic lines. As expected, the expression level of *Hl*WRKY1 was several folds higher than *Hl*WDR1, reflected its auto-activation activity [19]. The expression of the gene encoding *Hl*Myb1 regulatory protein was not increased, which was similar to the transcriptome data analysis. Among the structural genes involved in PF and BA biosynthesis pathways, the maximum enhancement in expression was observed in the case of *CHS*_H1. However, the distinct expression level of other structural genes associated with flavonol and anthocyanin pigmentation pathways was not observed, except for the *F3H* gene. The expression patterns of all the selected genes analyzed by qRT-PCR were consistent with the DEGs analysis (Fig. 6; Additional file 11: Table S5).

### Analysis of flavonoids content in WW-transgenic line

Hop leaves contain very low content of PF and BA compared to mature cones [81] and therefore, their quantification is difficult to analyze using HPLC method [82]. However, in order to evaluate the possible change in secondary metabolite content in leaves of WW-transgenic lines, we performed metabolite fingerprinting by HPLC. The contents of α-bitter acids (~ 60 fold) and xanthohumol (~ 40 fold) were enhanced significantly (Additional file 12: Figure S7) in leaves of WW-transgenic lines compared to WT hop.

## Discussion

Hop plants have been widely used in the brewing industry and practiced as traditional medicine since ancient times. Since hop extracts have beneficial and positive effects on human health and exhibit a wide range of biological activities with several other potential interesting applications, therefore, it has been the subject of systematic studies for more than three decades. The recent application utilizes crude spent hops extract as a botanical dietary supplement [83] and as an antifeedant activity to suppress stored*-*product insect pest populations [84]. For these reasons, conventional breeding and genetic engineering programs have been implemented in hop plant in efforts to enhance levels of biologically active compounds of interest [23, 85, 86]. The transcription factors have emerged as a promising candidate for genetic engineering owing to their role as master regulators of multiple target genes [26, 87]. In this context, our earlier study pertaining to *HlMYB*3 overexpression in hop demonstrated several folds enhanced accumulation of flavonoids and phloroglucinols accompanied by enhanced expression of *CHS*_H1, *CHI*, *F3’H*, *VPS* and *OMT1* genes [25]. Similarly, the heterologous expression of *Arabidopsis* transcription factor (*AtMYB12*) in tomato resulted in significant enhancement of flavonols and phenolics content in fruits by activating genes involved in the flavonoid biosynthesis pathway [28].

The *Hl*WRKY1 transcription factor shares significant homology to *Arabidopsis At*WRKY75 [19], which regulates phosphate acquisition and modulates root architecture [21], whereas *Hl*WDR1 transcription factor shows homology to Transparent Testa Glabra 1 (TTG1), encoding WD40 repeat transcription factor, which is involved in many aspects of plant development, regulation of flavonoid/anthocyanin biosynthesis, accumulation of seed storage reserves and trichome formation in leaves [22]. In this study, *Hl*WRKY1 and *Hl*WDR1 transcription factors were constitutively over-expressed in hop transgenic lines to enhance PF and BA content. Furthermore, we investigated for genome-wide alteration of steady-state transcript levels by comprehensive transcriptome profiling to build up information about the overall impact of constitutive expression of *Hl*WRKY1 and *Hl*WDR1 transcription factors over the morphological, developmental attributes and other related pathways of hop.

Transcript profiling and comparative transcriptome analysis with the aid of the high-throughput mRNA sequencing (RNA-Seq) technologies have frequently been used to identify networks of differentially expressed genes and genes expression patterns in several plant species. In this study, high-throughput sequencing generated more than 51 million high-quality reads, which were assembled into 23,666 unigenes. Subsequently, unigenes were functionally annotated using nr, GO, COGs and KEGG databases. The annotation results provide a valuable resource for further investigating the specific processes, pathways, and functions in the hop. Approximately 24% of unigenes were not annotated and could be considered as novel transcripts or alternative splice variants. The results of DGEs analysis suggested the alteration in gene expression level in WW-transformants and these genes were not only the components of PF and BA biosynthesis pathways but also belonging to other pathways involved in carbohydrate metabolism and lipid metabolism. Such modulation facilitates the enhancement of precursor and substrate molecules for flavonoid biosynthesis via central metabolic pathways connecting malonyl-CoA (product of carbon metabolism) and coenzyme A esters (product of lipid metabolism) with the plant flavonoid biosynthesis [88]. The up-regulation of *PAL* gene in WW-transformants line also suggested the enhanced flux of substrate towards the flavonoid pathway. The substrate channeling is a common event in cellular metabolism and such type of metabolic reprogramming involves a dramatic biosynthesis of transporters, which play a pivotal role in the efficient channeling of substrates ranging from organelles to the whole plant [89]. Nevertheless, we cannot rule out this possibility as genes encoding sugar, amino acid, lipid and ammonium transporters were differentially regulated in WW-transgenic hop plants. In this context, the integration of metabolomics, transcriptomics and proteomics data could be used as a future prospect to understand interactions between metabolites and genes/proteins and their relations in substrate channeling in flavonoid biosynthesis. Genes involved in photosynthesis (chlorophyll a/b binding proteins, chloroplastic protein, etc.) and stress response (pathogenesis-related protein: PR-1; PR-10, ethylene-responsive transcription factor 1B, TMV resistance protein N-like, ABC transporters etc.) were up-regulated. Several plant species exploit flavonoids as signaling molecules and their enhanced expression concurrently activates the expression of stress*-*related genes [30, 90, 91]. These observations indicate that increased flavonoids accumulation probably triggered the signaling cascades leading to the activation of stress-related genes in WW-transformants. The role of mitogen-activated protein kinase (MAPK) cascades via chromatin reprogramming by histone deacetylase 2 (HDAC2) in various stress response has been well documented in plants [92, 93], and therefore it is possible that modulation of MAPK cascades in WW transformants could cause the synergistic changes in HDAC2, which in turn through the chromatin reprogramming modulate the expression of genes associated with stress responses.

Several genes functioning in hormone biosynthesis and signaling pathways were differentially regulated in WW-transformants. In plants, auxin is an important growth hormone which regulates a wide array of growth and development processes [94]. The gene expression of the auxin-induced protein (X10a), IAA-amino acid hydrolase (ILR1) and phytohormone signaling component such as auxin-binding protein (ABP19a-like), were found to be up-regulated in WW-transformants and could be attributed to their better growth performance compared to WT-hop plants. The enhancement of secondary metabolites production through the process of metabolic reprogramming can modulate the expression level of various genes associated with phytohormone biosynthesis and signaling [95] and thus corroborate a plausible mechanism of modulation of genes associated with auxin and gibberellin acid biosynthesis and signaling pathway in WW transformants.

Among various modulators, light has been reported to be one of the most important environmental factors affecting flavonoid biosynthesis [96, 97]. Cryptochromes are blue, green and UV-A light flavoproteins photoreceptors and involved in photomorphogenesis, adaptive and growth processes, including biosynthesis of secondary metabolites, such as flavonoids in plants [98, 99]. Notably, in our RNA-sequencing data, cryptochromes interacting transcription factor TCP2 were up-regulated, suggesting that flavonoids levels appear to be sensitive to change prominently under the light in WW-transformants compared to WT hop. Similarly, the genes involved in flavonoid biosynthesis have been reported to be positively regulated by light in transgenic tobacco overexpressing *AtMYB111* transcription factor [29]. In our experiments, *Hl*WRKY1 expression level responded non-synergistically with that of *Hl*WDR1. The *WRKY* gene promoter consists of high frequencies of W-box *cis*-elements (WRKY binding site) and can autoactivate their own expression [100]. This fact explains the discrepancies associated with several fold higher transgene expression level *Hl*WRKY1 compared to *Hl*WDR1 and corroborated by our previous report relating the high frequency of the W-box motif on *Hl*WRKY1 promoter [101] and a state of high expression of *Hl*WRKY1 sustained by autoactivation [19]. The important finding of the present study is that WW-transgenic lines exhibited up-regulation of all the structural genes, namely *PAL*, *C4H*, *4CL*, *CHS*_H1, *PRT1*, *OMT1* and transcription factors *Hl*Myb2/*Hl*Myb3, *Hl*bHLH2, *Hl*WDR1 of MBW complex involved in PF and BA biosynthesis pathway. The up-regulation of structural genes involved in terminal steps of PF and BA biosynthesis was in accordance with our previous reports that *Hl*WRKY1 and *Hl*WDR1 transcription factor through independent or combinatorial activity drives the direct activation of *OMT1* and *PRT1* genes, and with the interaction with transcription factors of MBW complex drives the indirect activation of *CHS*_H1 gene of PF and BA biosynthesis pathways [19]. However, interestingly, we observed the relative up-regulated expression level of the structural genes *PAL*, *C4H*, *4CL* of the general phenylpropanoid pathway. Growing evidence suggests that the metabolite levels can regulate metabolic enzymes on various levels, from specific allosteric modulation to more complex transcriptional regulation [102]. Therefore, it is probable that enhanced substrate flux regulates the expression level of the PAL. In this study, novel transcription factors belonging to MYB, bHLH, WRKY families were found to be upregulated. The family members of these transcription factors are involved in the regulation of various biological processes, including signal transduction, secondary metabolism, development and stress responses [103, 104]. The changes in expression level of new MYB, bHLH, WRKY transcription factors either corroborated their regulatory role in early step gene activation of PF and BA biosynthesis pathway or a role in the development and stress responses, which are needed to ascertain and unravel through further experimentations. Furthermore, the expression analysis revealed the upregulation of flavanone 3-hydroxylase (*F3H*) gene without the modulation of genes involved in anthocyanin pigmentation and flavonol biosynthesis pathway. It seems that overexpression of *Hl*WRKY1 and *Hl*WDR1 may divert the enhanced flux of the common substrate (L-phenylalanine) more specifically towards PF and BA biosynthesis pathways compared to flavonols and anthocyanins biosynthesis pathways.

In this study, we conclude that *Hl*WRKY1 and *Hl*WDR1 overexpression lead to dramatic changes in genome-wide transcriptome with positive feed back impact on the expression levels of genes involved in PF and BA biosynthesis pathways. Our comprehensive study represents a valuable contribution towards strategies to analyze plausible changes in WW-transgenic lines and provides a novel contender gene for enhancing the PF and BA content in hop through genetic engineering or breeding program. The present study was conducted under greenhouse conditions during 2 years growing seasons of WW-transformant lines and WT hop plants. At the end of second year growing seasons, WW-transformant lines and WT hop plants have been reached a standard height but with inadequate flower initiation. In the coming year, WW-transformant lines and WT plants will be allowed to grow under contained field conditions to achieve typical flowering with maximum cone size and yield. Our future research aims at metabolite profiling of flavonoids, bitter acids, terpenes, fatty acids, sugars and differential expression analysis of genes using lupulin gland samples derived from the female cone of WW-transformants and WT hop plants to correlate the present results and evaluate the performance and accumulation of secondary metabolites in genetically modified hop in field-testing condition.

## Conclusion

In the present study, we conclude that overexpression of *Hl*WRKY1 and *Hl*WDR1 leads to up-regulation of structural and regulatory genes involved in prenylflavonoid and bitter acid biosynthetic pathways without any deleterious effect on the hop plants. Since several metabolic pathways are interconnected in order to allow an adequate regulation, thus possibly the perturbation of PF and BA biosynthesis pathways could lead to dramatic changes in the genome-wide transcriptome. With the availability of cones of WW-transformants, the detailed RNA sequencing and metabolite profiling will be performed to correlate and strengthen our present findings that *Hl*WRKY1 and *Hl*WDR1 genes could be served as potential contender genes to be used for secondary metabolome improvement programmes.

## Additional files

Additional file 1: **Figure S1.** Stereomicroscopic photograph showing the distribution of lupulin glands on the bracteole surface of a cone (A) and adaxial side of leaf surface (B) of Osvald’s cultivar of hop. (JPG 173 kb)

Additional file 2: **Table S1.** Primers used for cloning into plant vector, probes preparation, and qRT-PCR analyses. (DOC 62 kb)

Additional file 3: **Figure S2.** Regeneration of WW-transgenic plants of hop via *Agrobacterium*-mediated transformation of nodal explants (A), Phenotypic comparison of the growth of in vivo-grown WW-transformant and wild-type hop plantlets (B), Representative 2-year-old WW-transgenic and wild-type hop plant growing in the greenhouse condition (C), Leaf morphology of 2-year-old WW-transformant compared to wild-type hop plants (D) (Scale: 5 cm). (JPG 414 kb)

Additional file 4: **Figure S3.** Relative levels of transcription factor transgenes *HlWRKY1*, *HlWDR1* expression in the leaves of three independent lines of hop transformed with *Hl*WRKY1 and *Hl*WDR1 genes using vector WWpPCV91. RT-qPCR analyses were normalized using GAPDH as a house-keeping gene. The fold change of each gene was calculated by the 2^−ΔΔ*CT*^ method. *Statistically significant differences (*P* < 0.05); **significant at *p* < 0.01. (JPG 109 kb)

Additional file 5: **Table S2.** Morphological and physiological characteristics of the WW-transgenic plants compared to a wild-type plant of hop. (DOC 32 kb)

Additional file 6: **Table S3.** unigenes with significant BLAST X hits against the nr protein database. (XLS 2444 kb)

Additional file 7: **Figure S4.** Gene Ontology (GO) classifications of assembled unigenes (**A**) and differentially expressed genes in WW transformants compared with wild-type (**B**). The results are summarized in three main categories: Biological process, Cellular component and Molecular function. (JPG 468 kb)

Additional file 8: **Figure S5.** Histogram presentation of clusters of orthologous groups (COGs) classification of unigenes (A) and differentially expressed genes in WW-transformants compared with wild-type hop (B). (JPG 469 kb)

Additional file 9: **Table S4.** Transcription factors identified in the transcriptome of hop. (XLS 106 kb)

Additional file 10: **Figure S6.** Distribution of top 11 identified transcription factors from hop unigenes into transcription factor families. (JPG 165 kb)

Additional file 11: **Table S5.** The unigenes differentially expressed between the control and WW-transgenic plants of hop. (XLS 296 kb)

Additional file 12: **Figure S7.** HPLC analysis of menthanolic extracts of leaves of WT and WW-transgenic hop. Quantification (% DM) of (A) Gallic acid (phenolic acids), (B) α-bitter acids, and (C) xanthohumol was performed using their respective working standards. The graph shows values ± SD of three leaves from B11, B22, and B24 transgenic lines of the hop. (JPG 111 kb)

## Abbreviations

4CL: 4-coumaroyl CoA-Ligase
BA: bitter acids
C4H: cinnamate 4-hydroxylase
CHS_H1: “true” chalcone synthase
OMT1: O-methyltransferase 1
PAL: phenylalanine ammonia-lyase
PF: prenylflavonoid
PRT: prenyltransferase
qRT-PCR: quantitative real-time PCR, WT, wild-type
WW-lines: HlWRKY1 and HlWDR1 overexpressing lines
