# Supplementary figures and images for: Genome-wide transcriptome profiling of transgenic hop (Humulus lupulus L.) constitutively overexpressing HlWRKY1 and HlWDR1 transcription factors
Source: BMC Genomics. 2018 Oct 11;19:739. doi: 10.1186/s12864-018-5125-8 (PMC6180420; doi:10.1186/s12864-018-5125-8)

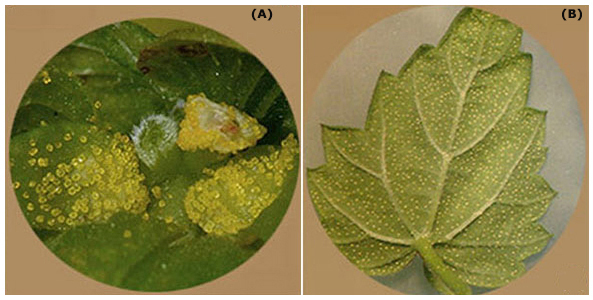

Supplement: Supplementary file 1 — Figure S1. Stereomicroscopic photograph showing the distribution of lupulin glands on the bracteole surface of a cone (A) and adaxial side of leaf surface (B) of Osvald’s cultivar of hop. (JPG 173 kb) [file 12864_2018_5125_MOESM1_ESM.jpg]

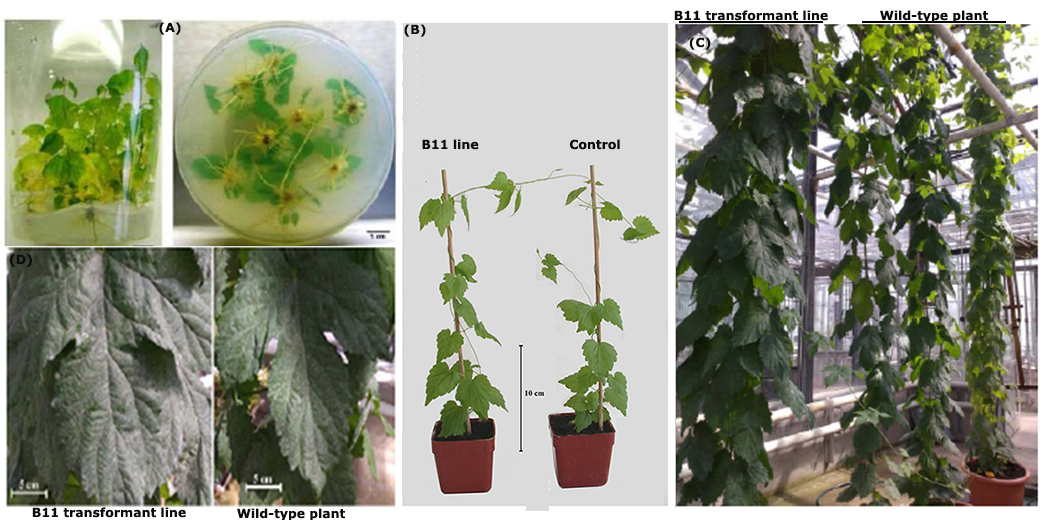

Supplement: Supplementary file 3 — Figure S2. Regeneration of WW-transgenic plants of hop via Agrobacterium-mediated transformation of nodal explants (A), Phenotypic comparison of the growth of in vivo-grown WW-transformant and wild-type hop plantlets (B), Representative 2-year-old WW-transgenic and wild-type hop plant growing in the greenhouse condition (C), Leaf morphology of 2-year-old WW-transformant compared to wild-type hop plants (D) (Scale: 5 cm). (JPG 414 kb) [file 12864_2018_5125_MOESM3_ESM.jpg]

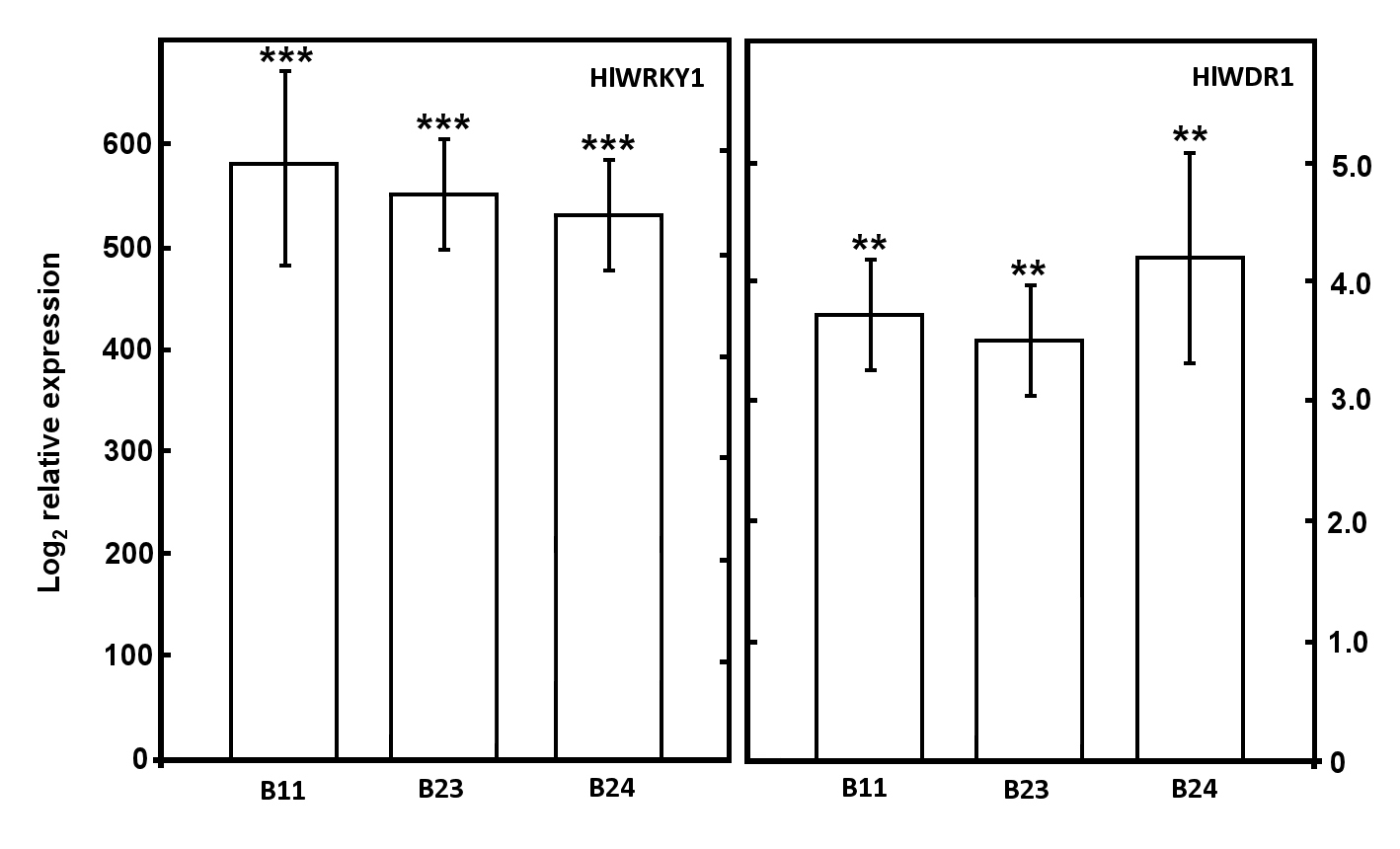

Supplement: Supplementary file 4 — Figure S3. Relative levels of transcription factor transgenes HlWRKY1, HlWDR1 expression in the leaves of three independent lines of hop transformed with HlWRKY1 and HlWDR1 genes using vector WWpPCV91. RT-qPCR analyses were normalized using GAPDH as a house-keeping gene. The fold change of each gene was calculated by the 2−ΔΔCT method. *Statistically significant differences (P < 0.05); **significant at p < 0.01. (JPG 109 kb) [file 12864_2018_5125_MOESM4_ESM.jpg]

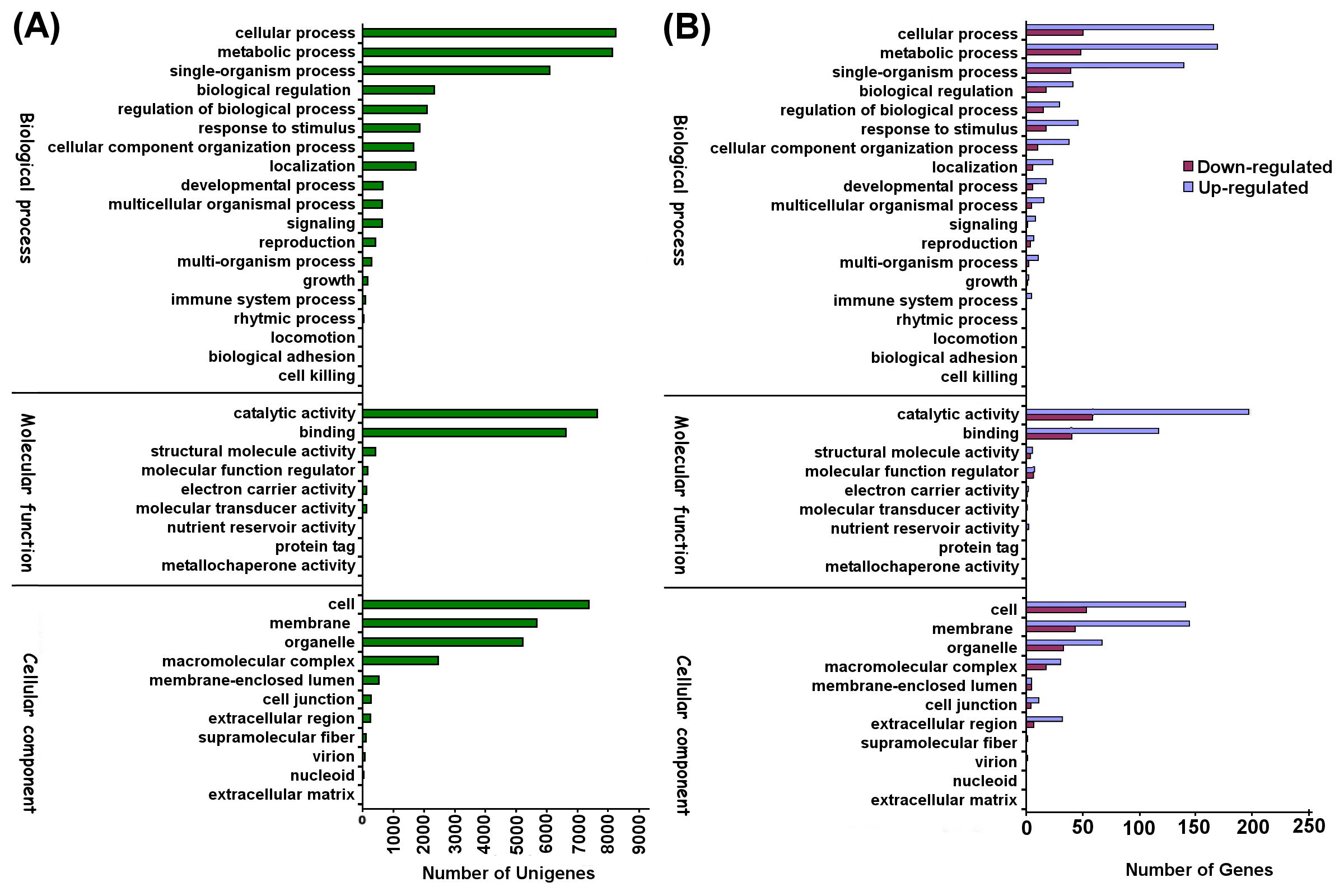

Supplement: Supplementary file 7 — Figure S4. Gene Ontology (GO) classifications of assembled unigenes (A) and differentially expressed genes in WW transformants compared with wild-type (B). The results are summarized in three main categories: Biological process, Cellular component and Molecular function. (JPG 468 kb) [file 12864_2018_5125_MOESM7_ESM.jpg]

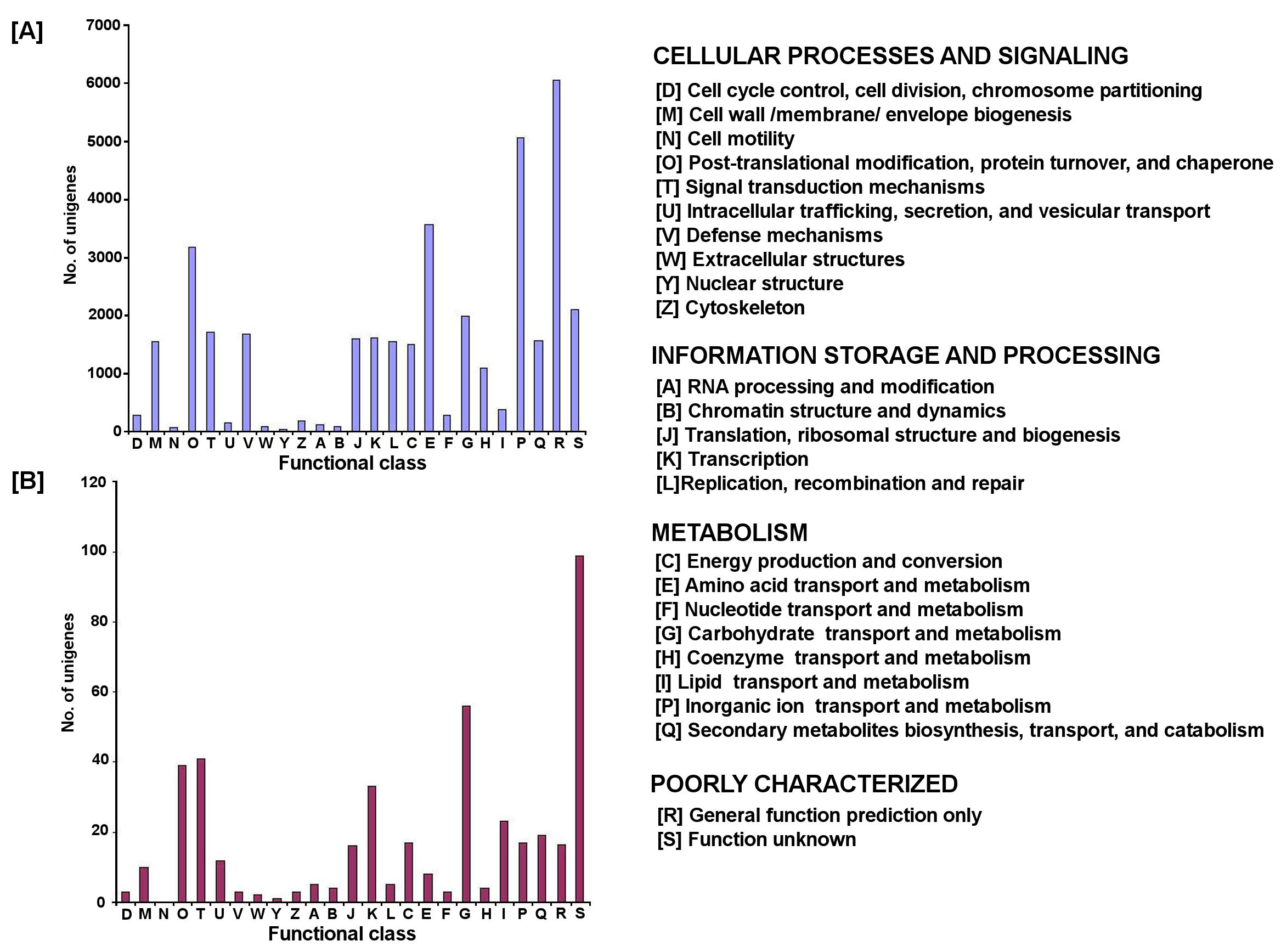

Supplement: Supplementary file 8 — Figure S5. Histogram presentation of clusters of orthologous groups (COGs) classification of unigenes (A) and differentially expressed genes in WW-transformants compared with wild-type hop (B). (JPG 469 kb) [file 12864_2018_5125_MOESM8_ESM.jpg]

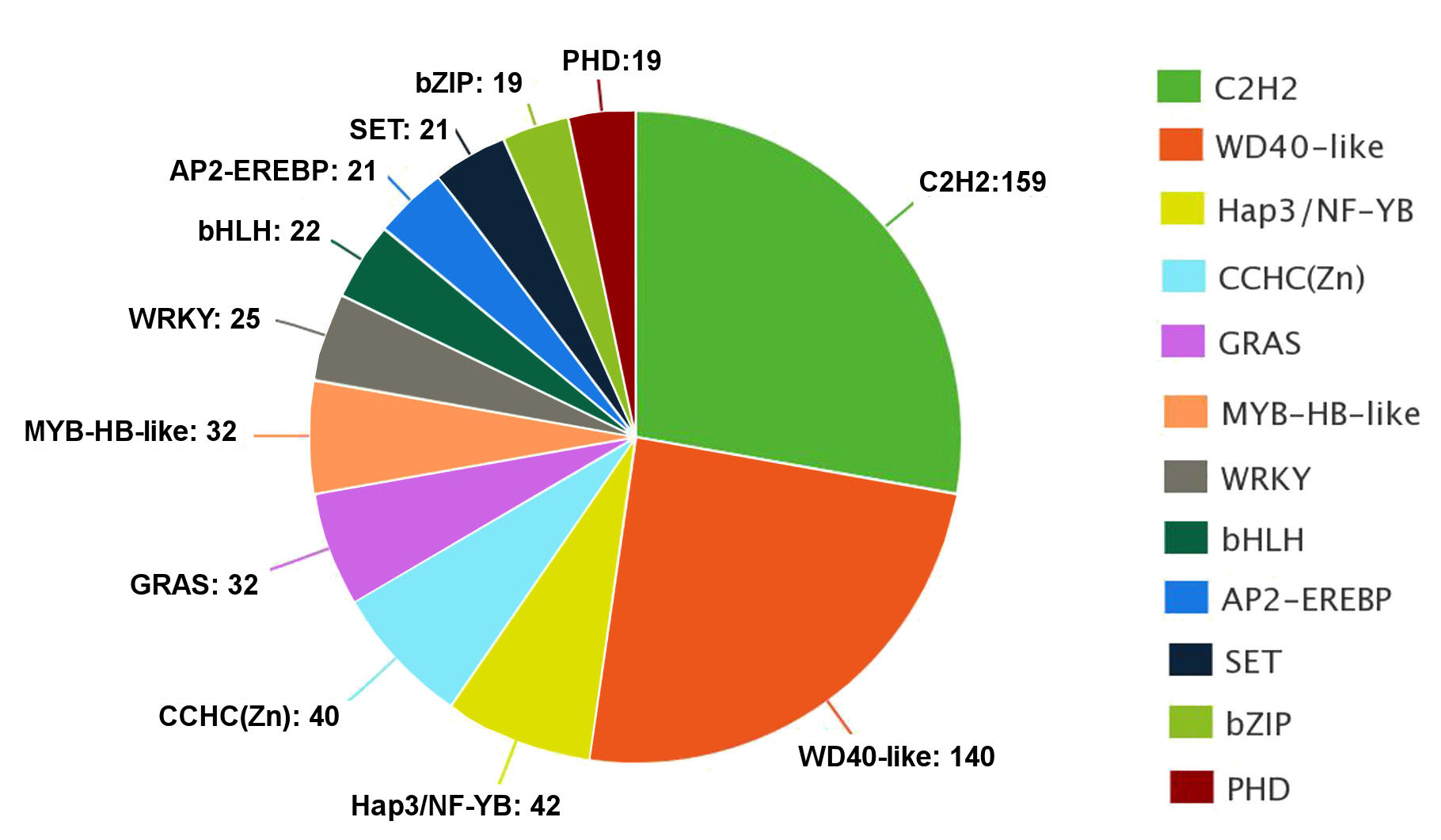

Supplement: Supplementary file 10 — Figure S6. Distribution of top 11 identified transcription factors from hop unigenes into transcription factor families. (JPG 165 kb) [file 12864_2018_5125_MOESM10_ESM.jpg]

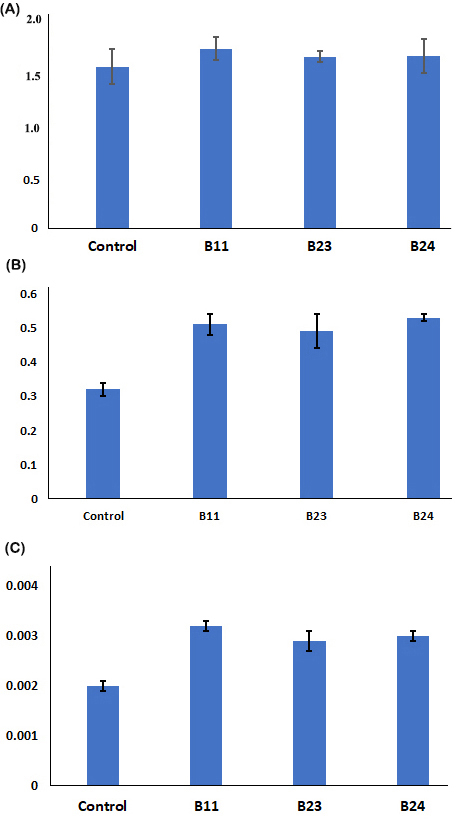

Supplement: Supplementary file 12 — Figure S7. HPLC analysis of menthanolic extracts of leaves of WT and WW-transgenic hop. Quantification (% DM) of (A) Gallic acid (phenolic acids), (B) α-bitter acids, and (C) xanthohumol was performed using their respective working standards. The graph shows values ± SD of three leaves from B11, B22, and B24 transgenic lines of the hop. (JPG 111 kb) [file 12864_2018_5125_MOESM12_ESM.jpg]
