# Supplementary material for: Genome-wide transcriptome profiling of transgenic hop (Humulus lupulus L.) constitutively overexpressing HlWRKY1 and HlWDR1 transcription factors
Source: BMC Genomics. 2018 Oct 11;19:739. doi: 10.1186/s12864-018-5125-8 (PMC6180420; doi:10.1186/s12864-018-5125-8)
Supplement: Supplementary file 2 — Table S1. Primers used for cloning into plant vector, probes preparation, and qRT-PCR analyses. (DOC 62 kb) [file 12864_2018_5125_MOESM2_ESM.doc]

**Table S1** Primers used for cloning into plant vector, probes preparation and qRT-PCR analyses

| **S.No** | **Primer Name** | **Sequence (5' - 3')** | **Purpose** |
| --- | --- | --- | --- |
| 1 | *Hl*WRKY1 Bam (Start) | aaGGATCCCCGCGGATGTCAGAGATGGAGATCCC | Vector cloning |
| 2 | *Hl*WRKY1 Bam (Stop) | aaGGATCCTTAAAAGGAAGTAGTGTAGATTTG | Vector cloning |
| 3 | *Hl*WDR1 Sal (Start) | aaGTCGACGGGCCCATGGAGAACTCGACGCAAG | Vector cloning |
| 4 | *Hl*WDR1 Sal (Stop) | aaGTCGACTCAAACTTTCAAAAGCTGC | Vector cloning |
| 5 | *Hl*WDR-PF | ATGGAGAACTCGACGCAAG | Probe preparation |
| 6 | *Hl*WDR-PR | TCAAACTTTCAAAAGCTGC | Probe preparation |
| 7 | HPT-F | TTCTGATCGAAAAGTTCGACAGCGTCTCC | Probe preparation |
| 8 | HPT-R | AATTGCCGTCAACCAAGCTCTGATAGAGTTG | Probe preparation |
| 9 | *Hl*WRKY1-F | AGATTGATCAGAGCTCCGACAGT | qRT-PCR |
| 10 | *Hl*WRKY1-R | CTTCCTCCATCTATAGCCATCATC | qRT-PCR |
| 11 | *Hl*WDR1-F | TTGCTTAGATTGGCTTGGAATAA | qRT-PCR |
| 12 | *Hl*WDR1-R | CCGGCTGAGCAGATATGTCTAT | qRT-PCR |
| 13 | HlMyb1-F | CTCAACTTGGCTCGGTTCTC | qRT-PCR |
| 14 | HlMyb1-R | CTGGTGTTCCCATTTGTTCC | qRT-PCR |
| 15 | *Hl*Myb2-F | TAGTGGGTCAGAGTACAGTGCTCAT | qRT-PCR |
| 16 | *Hl*Myb2-R | CAACCTCGAGAAGCTGCTGATA | qRT-PCR |
| 17 | *Hl*Myb3-F | GACGTCAACAGCAAGCAATTC | qRT-PCR |
| 18 | *Hl*Myb3-R | GGCCTCTGACGTGTCTGATG | qRT-PCR |
| 19 | *Hl*bHLH2-F | GACCAGCGGAGCGGGTTGAC | qRT-PCR |
| 20 | *Hl*bHLH2-R | CTCTGGCCGAGTTGACGGCG | qRT-PCR |
| 21 | PAL-F | CCGAAGTCTTGTCAGCCATT | qRT-PCR |
| 22 | PAL-R | TGGGGTGATGTCCTAAGAGC | qRT-PCR |
| 23 | C4H-F | CCACTGGAAGAAGCCAGAAG | qRT-PCR |
| 24 | C4H-R | TCTGCACCAAACGTCCAATA | qRT-PCR |
| 25 | 4CL-F | TCCGATAGCCTTAACGGTTG | qRT-PCR |
| 26 | 4CL-R | CCATAGCCCTGTCCAAGTGT | qRT-PCR |
| 27 | CHS_H1-F | ATCACTGCCGTCACTTTC | qRT-PCR |
| 28 | CHS_H1-R | AAATAAGCCCAGGAACATC | qRT-PCR |
| 29 | PRT1-F | ACAACAACAACAACACCTCTAACA | qRT-PCR |
| 30 | PRT1-R | CTTGCAGCTGAAAATTGATAAAG | qRT-PCR |
| 31 | OMT1-F | TAAAGGAACAGTGGTGGACGTTG | qRT-PCR |
| 32 | OMT1-R | ACCGCATCAGCACTAGGAATTGA | qRT-PCR |
| 33 | VPS-F | GTTATGCCGGTGGAAAA | qRT-PCR |
| 34 | VPS-R | CCGGCTTCCGTTACG | qRT-PCR |
| 35 | F3H-F | CACCTGAAACAGTCCCCAAT | qRT-PCR |
| 36 | F3H-R | GGGAGAAAACTCTCCGATCC | qRT-PCR |
| 37 | GAPDH-F | ACCGGAGCCGACTTTGTTGTTG | qRT-PCR |
| 38 | GAPDH-R | TCGTACTCTGGCTTGTATTCCTTC | qRT-PCR |

*Additional nucleotides are mentioned with small letters, attached restriction sites are underlined
