# Supplementary material for: Genome-wide transcriptome profiling of transgenic hop (Humulus lupulus L.) constitutively overexpressing HlWRKY1 and HlWDR1 transcription factors
Source: BMC Genomics. 2018 Oct 11;19:739. doi: 10.1186/s12864-018-5125-8 (PMC6180420; doi:10.1186/s12864-018-5125-8)
Supplement: Supplementary file 5 — Table S2. Morphological and physiological characteristics of the WW-transgenic plants compared to a wild-type plant of hop. (DOC 32 kb) [file 12864_2018_5125_MOESM5_ESM.doc]

**Table S2:** Morphological and physiological characteristics of the WW-transgenic plants compared to wild-type plant of hop.

| hop clone | height of the plants [m] * | number of nodes* | leaf area  [cm^2^]*** | glands per cm^2^ / leaf blade** | Total (a+b) chlorophyll content  [mg.g^-1^ fresh weight] |
| --- | --- | --- | --- | --- | --- |
| WT | 4.8 ± 0.76 | 32.33 ± 3.56 | 109.45 ± 23.68 | 93.51 ± 14.23 | 0.678 ± 0.13 |
| B11 | 5.2 ± 1.07 | 29.38 ± 2.72 | 191.18 ± 27.46 | 55.50 ± 8.75 | 1.323 ± 0.37 |
| B23 | 4.6 ± 0.82 | 31.04 ± 4.17 | 168.16 ± 20.95 | 42.47 ± 7.42 | 0.879 ± 0.53 |
| B24 | 4.9 ± 0.63 | 30.01 ± 2.97 | 145.24 ± 17.94 | 81.32 ± 9.26 | 1.134 ± 0.27 |

*To score height and nodes number, three two-year old plants of same developmental stage were evaluated.

**For evaluation of the leaf parameters, at least 48 leaves per each variant were scored. For each leaf usually two 1cm^2^ sectors were scored.

***Only trifoliate leaves from the nodes number 5 to 10 of same developmental stage were evaluated
